# Four-Dimensional Chromosome Structure Prediction

**DOI:** 10.3390/ijms22189785

**Published:** 2021-09-10

**Authors:** Max Highsmith, Jianlin Cheng

**Affiliations:** Department of Electrical Engineering and Computer Science, University of Missouri, Columbia, MO 65211, USA; chengji@missouri.edu

**Keywords:** genome, Hi-C, machine learning, computational biology, genomics

## Abstract

Chromatin conformation plays an important role in a variety of genomic processes, including genome replication, gene expression, and gene methylation. Hi-C data is frequently used to analyze structural features of chromatin, such as AB compartments, topologically associated domains, and 3D structural models. Recently, the genomics community has displayed growing interest in chromatin dynamics. Here, we present 4DMax, a novel method, which uses time-series Hi-C data to predict dynamic chromosome conformation. Using both synthetic data and real time-series Hi-C data from processes, such as induced pluripotent stem cell reprogramming and cardiomyocyte differentiation, we construct smooth four-dimensional models of individual chromosomes. These predicted 4D models effectively interpolate chromatin position across time, permitting prediction of unknown Hi-C contact maps at intermittent time points. Furthermore, 4DMax correctly recovers higher order features of chromatin, such as AB compartments and topologically associated domains, even at time points where Hi-C data is not made available to the algorithm. Contact map predictions made using 4DMax outperform naïve numerical interpolation in 87.7% of predictions on the induced pluripotent stem cell dataset. A/B compartment profiles derived from 4DMax interpolation showed higher similarity to ground truth than at least one profile generated from a neighboring time point in 100% of induced pluripotent stem cell experiments. Use of 4DMax may alleviate the cost of expensive Hi-C experiments by interpolating intermediary time points while also providing valuable visualization of dynamic chromatin changes.

## 1. Introduction

The three-dimensional (3D) conformation of the genome has been shown to play an important role in a variety of genomic processes, such as gene expression [1] genome replication [2] and gene methylation [3]. Various techniques have been developed for the analysis of 3D genome conformation, one of the most prominent being Hi-C [4,5], an improvement of the chromosome conformation capture (3C) technology, which applies the 3C principle in a genome wide, all-vs-all context. After alignment, Hi-C experiment reads are typically converted to contact matrices, which are then used to examine a plethora of higher order structural features, such as AB compartments [5], topological associated domains (TADs) [6,7,8], and 3D structural models [9,10,11,12,13,14].

Hi-C experiments can be categorized as bulk [15] or single cell [16]. Single cell Hi-C experiments have the advantage of inspecting only one cell, permitting inspection of structural variation; however, single cell experiments are often limited to low resolution due to their read coverage [17]. Conversely, bulk Hi-C experiments examine populations of cells, providing average contact information across millions of nuclei. Bulk Hi-C has the major advantage of high read coverage; however, it has been empirically demonstrated that bulk Hi-C samples can have large amounts of structural variation, meaning different cells in the population are positioned in varying orientations. To address this structural variation, some 3DModeling algorithms predict an ensemble of structures from a single bulk Hi-C contact map [18], while others predict consensus structures, which function as proxies for the average genomic conformation within the population [10,11,12].

As genomic sequencing has become cheaper, more researchers have begun to generate time-series Hi-C data. In such datasets, Hi-C contact maps are obtained along a time-dependent trajectory relating to genome function. Unfortunately, because 3C techniques are destructive to the cell in which they are applied, there are currently no time-series single cell Hi-C datasets. However, multiple datasets that use Bulk Hi-C are publicly available, including induced stem cell pluripotency [19] and cardiomyocyte differentiation [20]. While a plethora of meaningful and interesting observations have already been extracted from these datasets, analysis has been primarily constrained to comparing and contrasting individual points in the time series rather than the comprehensive analysis of four-dimensional (4D) chromatin conformation changes over multiple time points (i.e., three-dimensional conformation plus the fourth dimension of time). The need for novel 4D analysis has been identified as a critical and emerging area of research [21].

To address this need we introduce 4DMax, a maximum likelihood-based algorithm for predicting the transformation of chromatin conformation over the fourth dimension (time). By using spatial restraints derived from Hi-C contact matrices, we provide a tool that permits the generation of a predictive 4D video of chromatin conformational changes throughout the time series. 4DMax can be used to interpolate higher order chromatin features, such as the AB compartment and TADs, at times where no data is available while also providing valuable visualizations of chromosomal processes.

While many groups are now focused on modeling chromatin dynamics [22,23,24], to date, only one other group published a computational method for modeling 4D spatial transitions of chromatin exists, TADdyn [25]. Our 4DMax algorithm differs from TADdyn in three key ways.

Firstly, we utilized gradient descent optimization of a spatial restraint-based maximum-likelihood function, whereas the TADdyn approach utilized polymer modeling and steered molecular dynamics, followed by Monte Carlo-based simulated annealing.

Secondly, TADyn focused on small ~2 MB segments of the genome with emphasis on transcriptional dynamics, while our algorithm provided models of entire chromosomes. Our broader scope permitted meaningful analysis of higher-level structures, such as TADs and AB compartments across time.

Thirdly, we demonstrated that 4DMax can use generated models as an interpolation mechanism for predicting AB compartments and TADS at time points for which no Hi-C data has been gathered. 

The value of 4DMax is demonstrated through the construction of 4D models using contact maps derived from a mean-field simulated chromosomal looping process, as well as multiple real-time series Hi-C datasets. By studying the interrelation between contact maps, we are capable of identifying meaningful characteristics of the genomic process unavailable from analysis of only individual time points. Contact maps obtained through 4DMax interpolation frequently have high Spearman Correlation (0.66–0.81) with experimental data, often outperforming the average correlation between replicates (0.69). From these contact maps we successfully recover higher order conformational information, such as AB compartments from the predicted 4D structure, even at time points where true Hi-C maps are intentionally excluded. Out of the box 4DMax can easily be inserted into any analytic pipeline focused on time-series Hi-C analysis.

## 2. Results

### 2.1. Overview of the 4DMax Approach

**Figure 1:**
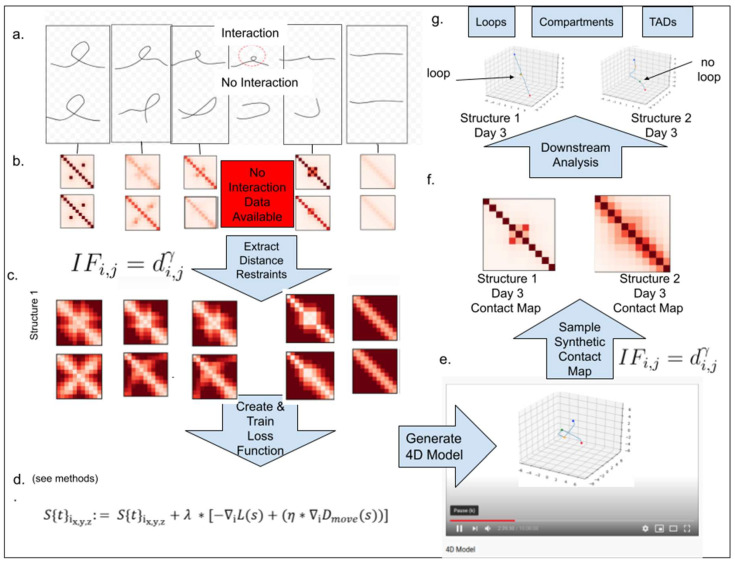
The graphic elucidates the 4DMax workflow using a simplified synthetic dataset as illustration. (**a**) Drawings of two potential chromosomal trajectories from identical starting and ending conformations. A significant contact at the center exists in structure 1 but not structure 2. (**b**) Contact maps obtained through synthetic Hi-C experiments on each day in the process. (**c**) Distance restraints derived from available contact maps. (**d**) Likelihood function for predicting 4D conformation. (**e**) Video of changing chromosome conformation. (**f**) Synthetic contact maps extracted at time of interest. (**g**) Different 3D structural conformations on day 3.

In Figure 1, we outline the overall framework of 4DMax. First, we gathered intrachromosomal contact matrices from different time points in a genomic process. Next, we converted contact matrices into spatial restraints using the relationship $D=IF^{\gamma }$, where D is distance, IF is interaction frequency, and γ is a negative exponent. This inverse relationship with distance and interaction frequency is frequently used in 3D modeling literature [10,14]. We then assigned a granularity g to denote the number of temporal snapshots where the spatial position of our 4D model would be identified. Using a maximum likelihood approach from probability theory, we then defined a likelihood function, which measures the agreement of our structure’s position at each time point, with temporally adjacent spatial restraints. We then initiated an unfolded structure using a uniform distribution over (0,1) and incrementally adjusted the structure’s position to maximize our likelihood function using a gradient ascent algorithm. After training, a smooth 4D Model was created, which can be visualized in movie format. From this 4DModel, we extracted synthetic Hi-C contact maps at time points of interest. We then used these extracted Hi-C contact maps for downstream Hi-C analysis, such as AB compartment classification and topologically associated domain (TAD) identification.

### 2.2. 4DMax Correctly Reconstructs Models of Synthetic Time Series Hi-C Data

We first created a simple, hypothetical chromosome and developed two theoretical structural progressions for the changing conformation of this chromosome. Both simulations were composed of 11 chromosomal bins of 50 kb width and evolved over a 6 day process. Each 4D structure began and ended identically, the initial chromatin state being in a looped formation and the final state being fully elongated. The two structures differed in their respective paths taken from their initial and final states. In structure 1, the loop unraveled as if pulled on both ends, while in structure 2 the loop swung open (Figure 1a). As a consequence of these differences in paths, on day 3, there was strong interaction between bins 4 and 6 on structure 1, but no such interaction existed on structure 2. 

We first defined contact maps for each of the six time points on both structures (Supplementary Material Figure S1) and used these contacts as inputs to 4DMax to generate novel 4D structures (Supplementary Video S1). We then simulated Hi-C experiments at the six time points using the generated structure and obtained contact maps with the above 0.95 Pearson Correlation (PCC) with corresponding input contact maps (Supplementary Material Figure S2). Furthermore, visual inspection of the two generated videos accurately displayed the unique behaviors of unraveling and swinging open, as previously described (Supplementary Video S1).

We tested the effectiveness of 4DMax in capturing 4D movement and predicting 3D position at time points where contact map information was unavailable. We ran four experiments for each synthetic structure, excluding contact maps for days 1, 2, 3, and 4, respectively. The PCC values between original synthetic Hi-C maps and their corresponding interpolations remained high, ranging from 0.82 to 0.99 (Supplementary Material Figure S2). Visually, we continued to observe the expected unraveling and swinging behaviors in each 4D video, even with excluded data (Supplementary Video S1).

### 2.3. 4DMax Predicts Smooth 4D Models of Induced Pluripotent Stem Cell Differentiation in Mice

**Figure 2:**
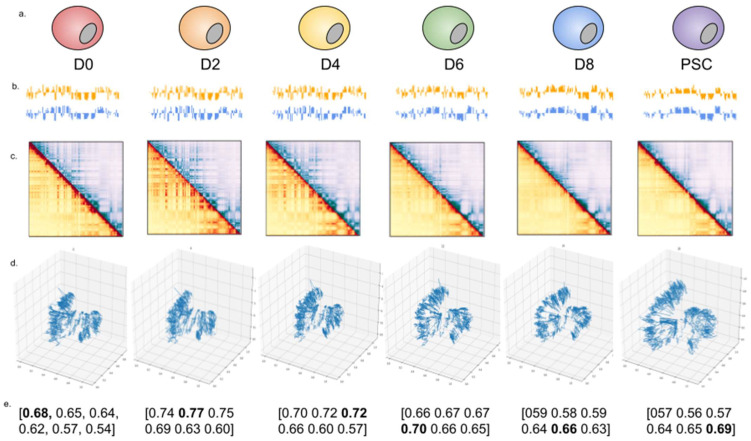
Diagram of outputs. (**a**) Outline of the different stages of the iPSC dataset of Mus Musculus. (**b**) AB compartment vectors of (orange) original and (blue) reconstructed contact maps. (**c**) 50 kb contact map of chromosome 13 by time, using (orange) original and (blue) reconstructed contact maps. (**d**) 4DMax prediction of structural conformation of chromosome 13 at time. (**e**) Spearman correlation between the above reconstructed contact map and the real contact maps at each time point.

We applied 4DMax to a 10-day time series Hi-C dataset of induced stem cell pluripotency in mice [19]. We used intrachromosomal Hi-C contact maps from day 0 (Beta), 2, 4, 6, 8, and 10 (PSC) at 50 kb resolution. We selected a granularity of 21, ensuring that each time point for which real data was available occurred within the time interval partition. 4DMax successfully produced smoothly changing structures for each chromosome (Figure 2c, Supplementary Video S2). We frequently observed a decrease in compression of 4D models as the induced pluripotency process progressed (Figure 2c, Supplementary Video S2). The 4DMax predictions for chromosomal position at the input times showed high similarity to 3D structures generated by previously built state-of-the-art 3D modeling algorithms with average SRC = 0.76 and PCC = 0.75 (Supplementary Material Figure S3).

Using the 4DMax predictions, we then simulated Hi-C experiments (Figure 2d, Supplementary Material Figure S4) at each of the input time points to obtain synthetic Hi-C maps. We refer to these contact maps as reconstructed contact maps. We compared these reconstructed maps to their corresponding real contact maps and observed high SRC values ranging (0.53–0.82) (Supplementary Material Figures S7 and S10). These values were consistently higher than the similarities seen between contact maps on days 0 and 10 (0.46–0.68) (Supplementary Material Figure S5). 

### 2.4. 4DMax Preicts Smooth 4D Models of Cardiomyocyte Differentiation in Humans

To verify the effectiveness of varied Hi-C datasets, we also applied 4DMax to a 14-day time series Hi-C dataset of cardiomyocyte cell differentiation at 500 kb resolution. The cardiomyocytes dataset contained Hi-C contact maps assayed at irregularly timed intervals on days 0, 2, 5, and 14. We built 4DModels with a granularity of 15, ensuring that each time point for which real data was available occurred within the time interval partition, preserving the uneven timing of the contact maps (Supplementary Material Figure S6). 4DMax again produced fluidly changing 4D models (Supplementary Video S3). We then simulated Hi-C experiments to obtain synthetic contact maps from the 4D model at the four input times and observed SRC values ranging from 0.54 to 0.92 between synthetic maps and their correspondingly-timed real Hi-C data (Supplementary Material Figure S9). We compared 4DMax reconstructed contact maps to real contact maps across all permutations of input times and observed that SRC values are highest with corresponding times in 93.2% of the reconstructions, indicating that the high correlation between real and reconstructed maps is significant relative to other Hi-C contact maps.

### 2.5. Interpolation of Time Series Hi-C Data Using 4DMax Generated Models Show High Consistency with Experimental Hi-C

**Figure 3:**
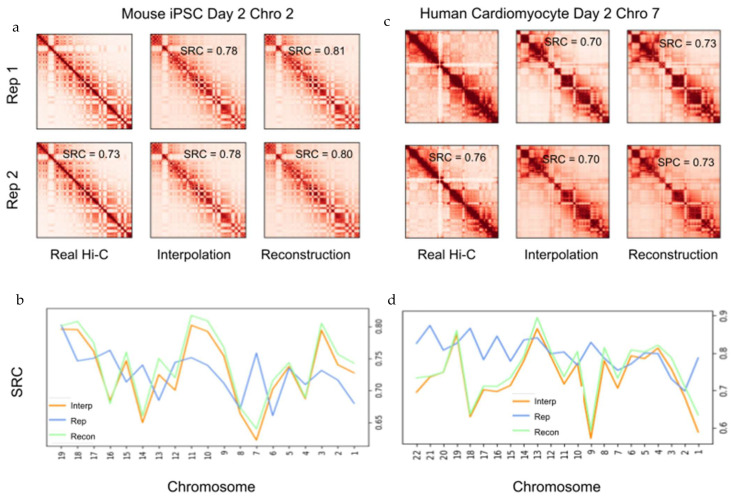
Example contact map comparison. (**a**) Contact maps of iPSC on day 2 chromosome 2 from Real Hi-C, 4DMax reconstruction, and the 4DMax day 2 agnostic interpolation model, using 50 kb matrices. (**b**) SRC of iPSC contact maps relative to Real Hi-C for each chromosome on day 2. (**c**) Contact maps of cardiomyocyte data on day 2 chromosome 7 from Real Hi-C, 4DMax reconstruction, and the 4DMax day 2 agnostic interpolation model using 500kb matrices (**d**) SRC of cardiomyocyte contact maps to Real Hi-C for each chromosome on day 2.

To evaluate the rigidity of 4DMax in its prediction of chromosomal position at time points between available contact maps, we ran four experiments on each chromosome, where we generated 4D models of the iPSC dataset while excluding Hi-C data for individual time points: D2, D4, D6, D8. We call these models the “iPSC Interp models”. The iPSC Interp models show high similarity to 4D models generated by the complete iPSC dataset (SRC > 0.99 in all chromosomes besides 1,4,5 PCC > 0.96), indicating the algorithm’s resilience to missing Hi-C data (Supplementary Material Figure S14, Supplementary Video S4). We then ran synthetic Hi-C experiments on the iPSC Interp models at the time point for which their data was excluded to obtain contact maps, which we call ‘Interpolation Contact Maps’. These Interpolation Contact Maps differed from the previously described Reconstructed Contact Maps, in that they were predicted using a 4DMax model that was not provided Hi-C contact information for the time being inspected; consequently, the maps were interpolations predicted by the 4DMax model. We compared these interpolated contact maps to corresponding real Hi-C contact maps and found high correlation with mean SRC = 0.73 with values ranging from 0.66 to 0.84 (Figure 3a,b). These results indicate that 4DMax was effective at predicting intermittent structures for time points where no Hi-C data was available. To further ensure the utility of 4DMax, we compared 4DMax interpolated contacts to contact maps derived from simple numerical interpolation of contact maps at adjacent time points. We can see that 4DMax outperformed this naïve interpolation in 87.4% of the instances (Supplementary Material Figure S20). 

We also performed interpolation experiments using the cardiomyocyte [20] dataset, where we excluded Hi-C input data on day 2. We refer to the resultant 4D models as the “Cardio Interp models”. The Cardio Interp models showed high correlation to 4D models generated, using the complete cardiomyocyte dataset (Supplementary Material Figure S15, Supplementary Video S5). We obtained synthetic Hi-C contact maps on day 2 from the Cardio Interp Model and compared these interpolation maps to the real day 2 Hi-C contact maps and found SRC values ranging from 0.57 to 0.87 (Figure 3c). In six of the chromosomes (28%), our interpolation showed higher correlation to the real Hi-C map than a biological replicate (Figure 3d, Supplementary Material Figure S8). These results indicate versatility in the time-series datasets, for which our 4DMax algorithm could effectively interpolate Hi-C data.

### 2.6. 4DMax Correctly Preserves and Predicts AB Compartment Assignment

**Figure 4:**
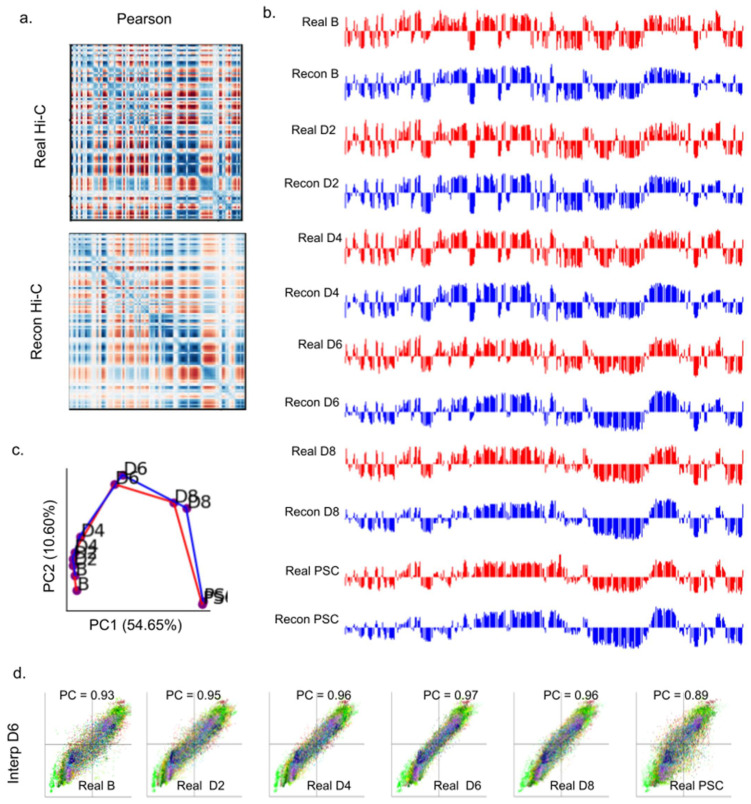
Analysis of AB compartment features of 4DMax-generated contact maps. (**a**) Pearson correlation matrices of chromosome 13 day 2 using Real Hi-C and synthetic contact maps obtained from the 4DMax model. (**b**) AB compartment vectors from chromosome 13 (red) real Hi-C data (blue) synthetic contact maps obtained from the 4DMax model. (**c**) Trajectory curve of the two largest principal components (red) real Hi-C (Blue) Reconstructed Hi-C. (**d**) Scatter plot of 100 kb binned AB compartment vectors, where x value is bins Real Data PC1 value and y value is interpolated contact maps PC1 value.

A primary value of Hi-C data is its utility in illuminating higher order structural features of chromatin [4]. One of the most prolific of these structural features are megabase scale subnuclear compartments, called AB compartments [5]. Regions of the genome were assigned to either compartment A or compartment B. The A compartment was associated with gene activity and euchromatin, while the B compartment was associated with inactive heterochromatin. The AB compartment assignment is known to change significantly during the iPSC process. AB compartment assignment can be derived by principal component analysis (PCA) of Pearson correlation matrices derived from Hi-C contact maps (Methods). We first performed comparative AB compartment analysis on real Hi-C contact maps and contact maps reconstructed from iPSC full models. We observed high visual similarity between Pearson correlation matrices of reconstructed and corresponding real Hi-C data across all chromosomes and time points (Figure 4a, Supplementary Material Figure S18). 

The iPSC dataset has previously been shown to undergo pronounced changes to compartmental organization as time progresses. Visually, we observed a high similarity between Reconstructed and Real AB compartment vectors at each point in the time series (Figure 4c, Supplementary Material Figure S13). We quantified this progression by treating AB compartment vectors as input vectors to PCA to obtain trajectory curves for each chromosome (Figure 4b, Supplementary Material Figure S11). The trajectories of Real and Reconstructed compartments matched one another closely. These analyses indicate that the 4D models generated by 4DMax maintained the higher order information needed for AB compartment analysis.

We also compared the AB compartment profiles of our interpolated iPSC matrices to AB compartment profiles of real Hi-C contact maps (Figure 4d, Supplementary Materials S12). In all four models, we saw PCC values greater than 0.96. Furthermore, when comparing interpolated AB compartment profiles to the AB compartment profiles of real Hi-C contact maps across all times in the iPSC process, we found the highest correlation at the interpolated time points (Figure 4d, Supplementary Materials S12). For example, we built an interpolation model for the 4D chromatin structure, excluding contact information on day 6, instead only showing the algorithm contact information for days 0, 2, 4, 8, and 10. 4DMax then made predictions for the chromosomal conformation on day 6. The output prediction for chromosomal conformations on day 6 were more similar to the real contact matrices on day 6 (0.97) than they were to any of the contact maps that the algorithm was exposed to (D0 = 0.93, D2 = 0.95, D4 = 0.96, D10 = 0.89). This trend was consistent across all interpolation models and is crucial as it indicates that 4DMax accurately predicted changes to AB compartment profiles, rather than simply obtaining high correlation due to maintained AB compartment profiles between adjacent time points.

### 2.7. 4DMax Correctly Preserves and Predicts TAD Border Positioning

**Figure 5:**
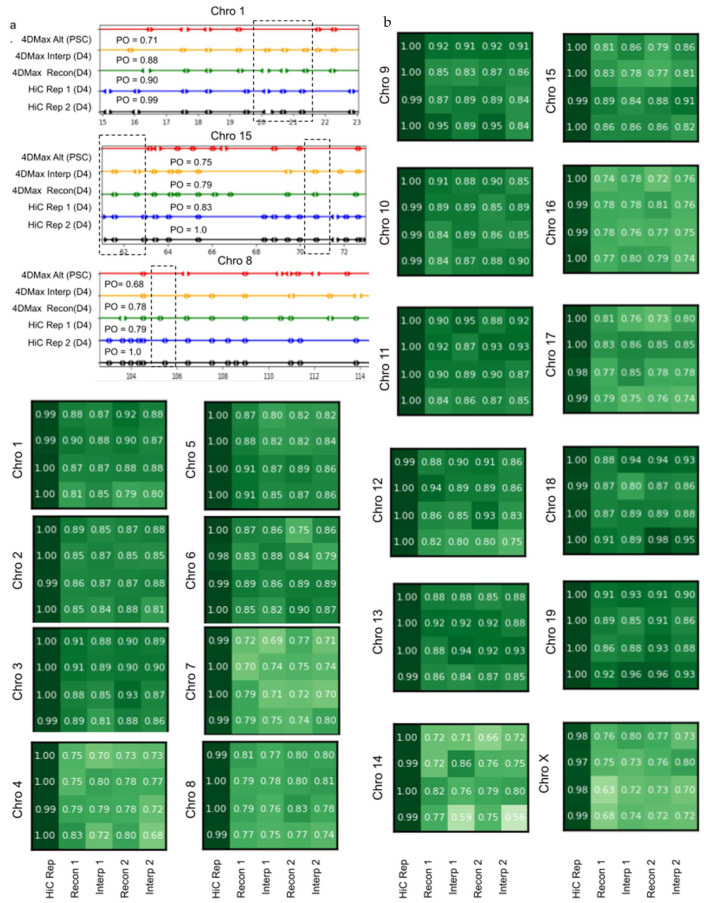
HiCTool identified topologically-associated domains. (**a**) Select images of TAD boundaries on (black) Real Hi-C replicate 1, (blue) Real Hi-C replicate 2, (green) 4DMax Reconstructed Map, (orange) 4DMax Interpolated Hi-C Map, and 4DMax Recon Map at a different time point. PO metric quantifies the percent of TAD boundaries found within 0.5 Mb of a boundary identified in Hi-C replicate 1. (**b**) PO of Interpolated and Reconstructed 4DMax TAD positions for both replicates across all chromosomes.

Another prolific use of Hi-C data is the identification of topologically associated domains (TADs). We used the Hi-C analysis tool HiCtool to identify TADs from contact maps in the iPSC dataset. We then used the HiCtool to identify TADs with synthetic contact maps derived from 4DMax reconstruction and interpolation models (Figure 5a,b). We observed high similarity in TAD profiles of reconstructed synthetic maps and Real Hi-C contact maps with a mean percent overlap of 84% and a peak of 95% on chromosome 9. We also observed high similarity in TAD profiles of interpolated synthetic maps and real Hi-C contact maps with a mean percent overlap of 83% and a peak of 96% on chromosome 11.

### 2.8. 4DMax Completes in Tractable Time for Human and Mouse Chromosome Construction

**Figure 6:**
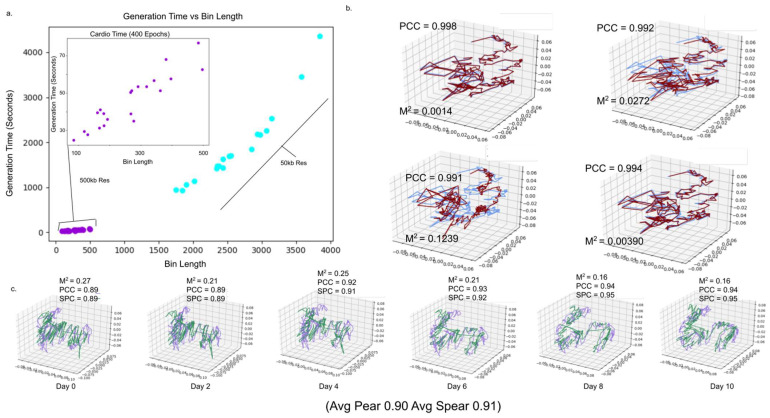
Evaluation of run times and computational stability. (**a**) Scatter plot of chromosome bin lengths and time to completion using 400 epoch (purple) 500 kb resolution chromosome and (blue) 50 kb resolution chromosomes. (**b**) 3D plot of predicted cardiomyocyte chromosome 10 on day 5 with varying granularity values. Spearman correlation Mean Squared distance compares (blue) granularity 15 structure to higher granularity structures (red). (**c**) 3D plots comparing (purple) 50 kb resolution chromosome 1 to (green) 500 kb resolution iPSC chromosome 1 on each day in time series.

The 4DModel generation time was determined by three parameters: training epochs, granularity and bin quantity. Run time scaled linearly with the number of training epochs (Supplementary Material Figure S16). Empirically, we observed 400 epochs as sufficient to obtain organized and consistent conformational changes in 4D models for both datasets (Supplementary Video S6). Granularity, defined as the number of tracked discrete time points in the interval, also impacted run time linearly. (Supplementary Material Figure S16). Bin quantity, defined as the number of discrete spatial points tracked per time point, was dependent on chromosomal length and resolution. We observed super linear growth of time as bin quantity increased (Figure 6a; Supplementary Material Figure S17). Using a single GTX 1080 Ti graphics card, 4DMax constructed 4DModels of 500 kb resolution chromosomes in a matter of minutes and took under 1.5 h to generate models from 50 kb resolution chromosomes.

### 2.9. 4DMax Predictions Remain Stable against Change in Time Point Granularity

We compared the 4D structures of the same chromosomes in the cardiomyocyte dataset with varying granularity of 15, 29, 43, 57, and 71. For each granularity comparison, we used the average correlation between time points present in both structures (Figure 6b, Supplementary Video S7). We saw minimal discrepancies between our maximal and minimal granularity values, with the average correlation (PCC = 0.90; SRC = 0.94) reaching as high as (PCC = 0.94, SRC = 0.99) on chromosome 9. These results indicate stability to changes in granularity.

### 2.10. 4DMax Predictions Remain Stable to Variation in Hi-C Contact Matrix Resolution

We compared the 4D structures of the same chromosomes in the iPSC dataset at 500 kb and 50 kb resolution. The structures from 50 kb resolution were reduced to 500 kb by averaging the position of every 10 consecutive spatial points (Figure 6c). The average correlation between structures remained high (SRC = 0.84, PCC = 0.83), reaching (SRC = 0.96, PCC = 0.97) for chromosome 8. This indicates consistent 4DModel predictions across varying resolutions.

## 3. Discussion

Here, we present 4DMax, a method used to examine time-dependent dynamics of chromatin during genomic processes. 4DMax is the first tool of its kind to provide comprehensive chromosome wide predictions of 4D dynamics. By converting contact maps at select times into spatial restraints, using these restraints to build a likelihood based objective function, and providing optimization with gradient ascent, 4DMax constructs smooth 4DModels.

The 4DMax framework makes multiple simplifying assumptions, which should be considered in the evaluation of results. 

First, our model assumed that observed contacts were drawn from gaussian probability distributions. While this assumption has been applied to genome modeling in previous literature [11], Hi-C data can be highly stochastic and may not follow the selected distribution. 

Second, because there are no existing time-series, for single cell Hi-C experiments, our model makes predictions on bulk Hi-C data. This has a major disadvantage in that bulk Hi-C data can contain high amounts of structural variability. Because our model predicts a single consensus structure, rather than multiple structures, our model likely represents a convenient proxy for an average model rather than a realistic model of the dynamics for any individual chromosome.

Thirdly, both the iPSC and Cardiomyocyte datasets are time-series data, with multiple days between samplings. Previous literature has demonstrated that chromosome folding is modulate during cell cycle development [26], meaning that there are conformational changes that occur on smaller timescales than those viewed in our consensus model.

Even with these simplifying assumptions, we demonstrate strong evidence for utility in the predictions made by 4DMax. We validate the effectiveness of 4DMax in predicting 4D conformations using both synthetic chromosomal conformations and real-time series Hi-C datasets from mice and humans. From these visualizations, we often observe pronounced changes to the positioning of chromosomes over time, such as the progressive decompression of mice chromosomes as they become pluripotent. 

In addition to the valuable visualizations, 4DMax accurately predicts chromosome position at time points where data is excluded from the 4DMax algorithm. The interpolated maps from 4DMax frequently show higher similarity to true contact maps at their corresponding time than to true contact maps at adjacent times presented to the model. This is particularly promising because it indicates that the high similarity of retrieved biological features is not a product of low chromosomal structural change in temporal segments of the time series, but rather that novel inferences are being made to the actual position of the chromosome at times where no hi-c data is available. Given these findings, 4DMax could be used by other labs as a preliminary substitute for expensive Hi-C experiments when examining a genomic process over time. 

4DMax is easily integrated into any time series Hi-C pipeline. Our model stability experiments show computational stability to the variation of parameters, such as contact map resolution and granularity, while maintaining a sufficiently short run time. The structures generated by 4DMax showed high correlation to input contact matrices, and the synthetic contact maps derived from predicted 4DMax structures frequently had high correlation with real contact maps, even at times where no contact map data was presented to the model. 4DMax-derived contact maps retained biologically relevant higher order features, such as AB compartment and TAD placement.

Despite these promising results, the time scale of all real Hi-C datasets tested was in the order of days, therefore it was possible that significant changes to chromosome conformation may have occurred at smaller intervals not captured by existing data. To address this concern in the future, 4DMax will have to be applied to future time series Hi-C datasets with smaller time intervals and additional assays for validation of conformation, such as Capture Hi-C and microscopy data.

## 4. Materials and Methods

### 4.1. Description of 4DMax Algorithm

4DMax is intended for researchers interested in inspecting the changing structural conformation of the genome over the duration of a dynamic biological process. We assumed that the biological process occurs over a time Interval I = (0,T), where 0 is the start of the process and T is the last point in the process. I is theoretically continuous but is computationally represented as a collection of equally spaced times between 0 and T. The quantity of points in the computational representation of I is referred to as the granularity, g. We represented a chromosome’s movement over a time interval as a collection of *n* points in 4D space. Let S be a 4D chromosome structure, where S [27] denotes a structure’s spatial position at a given time. Let $S_{i}$ denote the i-th bin of our structure, meaning $S_{i}${t} denotes the position of the i-th bin of structure S at time t. Because our structures exist in 4D space, $S_{i}t$ is represented by an $\left(x,y,z\right)\;\in R^{3}$ coordinate and S $\in \;\left(R^{3}\times I\right)$.

We used a likelihood function as a loss function to compute chromosome conformation from the contact maps. Let $H=\{H_{\tau }\},$ where $H_{\tau }$ is the Hi-C contact map at time τ∈T and T is the collection of time points for which we had available contact maps. Note T∈I. The likelihood of a structural conformation S can be modeled as the product of the probability of the observed Hi-C contact maps H, conditioned on S{t}.

(1) $$L\left(s\right)\;=\underset{t}{\prod }P(H|S\left\{t\right\})$$

PH(S|t} can be modeled as the product of the individual distances in H conditioned on S by assuming that each constraint is independent. By assuming that each constraint $H_{i}\in \;H$ is conditionally independent of other constraints, we rewrite the likelihood as:

(2) $$L\left(s\right)\;=\underset{t}{\prod }\underset{i}{\prod }P(H_{i}|S\left\{t\right\})$$

Because our Hi-C samples were taken some point during the biological process being observed, we know that T ∈ I; however. if we select a high granularity for I, there are certain t∈I such that t ∉ T. Thus, we can separate our L(s) by

(3) $$L\left(s\right)\;=\underset{t\in T}{\prod }\underset{i}{\prod }P(H_{i}|S\left\{t\right\})\underset{t\notin T}{\prod }\underset{i}{\prod }P(H_{i}|S\left\{t\right\})$$

Because the logarithmic function is monotone, we can take the logarithm of L(s) without the argmax changing, yielding

(4) $$L\left(s\right)\;=\underset{t\in T}{\sum }log(\underset{i}{\prod }P(H_{i}|S\left\{t\right\})+\underset{t\notin T}{\sum }log(\underset{i}{\prod }P(H_{i}|s\left\{t\right\}))$$

When t∈T, we assume that the observed contact maps are drawn from a gaussian probability distribution:

(5) $$P(H_{i}\left\{t\right\}|S\left\{t\right\})\;∼\;\frac{1}{\sigma \sqrt{2\pi }}exp(\frac{-1}{2\sigma^{2}}{\left(D_{i}\left\{t\right\}\;-\;H_{i}\left\{t\right\}\right)}^{2})$$

where $D_{i}\left\{t\right\}$ is the euclidean distance between the pair of regions in our structure S, computed from (x,y,z) coordinates, and σ is the standard deviation of the gaussian distribution. By assumption of normal distribution, we know:

(6) $$\sigma =\sqrt{\frac{\sum_{i}\left(H_{i}\;-\;D_{i}\right)2}{n}}$$

Using algebra, we can manipulate Equation (5) to resemble a component of the first right-hand summation term in Equation (4), as shown in Equation (7).

(7) $$\underset{i}{\prod }P(H_{i}|S)={\left(\frac{1}{\sigma \sqrt{2\pi }}\right)}^{n}exp(\frac{-1}{2\sigma^{2}}\overset{n}{\underset{i}{\sum }}{\left(D_{i}\left\{t\right\}\;-\;H_{i}\left\{t\right\}\right)}^{2})$$

Thus, by taking the logarithm of both sides of (7), we obtain (8).

(8) $$log(\underset{i}{\prod }P(H_{i}|S))=\frac{\sum_{i}\left(H_{i}\left\{t\right\}\;-\;D_{i}\left\{t\right\}\right)}{2\sigma^{2}}-nlog\left(\sigma \right)$$

We can substitute Equation (6) for Equation (8) to remove all dependence on σ and obtain

(9) $$log(\underset{i}{\prod }P(H_{i}|S)=\frac{n}{2}-log(\sqrt{\frac{\sum {\left(H_{i}\left\{t\right\}\;-\;D_{i}\left\{t\right\}\right)}^{2}}{n}})$$

When t∉T, we assume

(10) $$a_{1}\left(t\right)\;=\;max\;\tau \;\in \;S_{\tau }\;where\;\tau <t\;a_{2}\left(t\right)\;=\;min\;\tau \;\in \;S_{\tau }\;where\;\tau >t\;w_{1\left(t\right)}=\frac{t\;-\;a_{2}\left(t\right)}{\left|a_{1}\left(t\right)-a_{2}\left(t\right)\right|},\;w_{2\left(t\right)}=1-w_{2}$$

and assume that

(11) $$log(\underset{i}{\prod }P(H_{i}|S\left\{t\right\}))=w_{1}\left(t\right)\;*\;log(\underset{i}{\prod }P(a_{1}\left(t\right)|S\left\{t\right\}))+w_{2}\left(t\right)\;*\;log(\underset{i}{\prod }P(a_{2}|S\left\{t\right\}))$$

Using Equations (7) and (11), we obtain

(12) $$\underset{i}{\prod }P(H_{i}|S\left\{t\right\})=w_{1}\left(t\right)[\frac{n}{2}\;-\;log(\sqrt{\frac{\sum {\left(a_{1}\left\{t\right\}\;-\;D_{i}\left\{t\right\}\right)}^{2}}{n}})]\;+\;w_{2}\left(t\right)[\frac{n}{2}\;-\;log(\sqrt{\frac{\sum {\left(a_{2}\left\{t\right\}\;-\;D_{i}\left\{t\right\}\right)}^{2}}{n}})]$$

Thus, by substituting Equations (8) and (12) into the first and second terms of (4), we obtain a well-defined likelihood function whose variables are either fixed values, such as Hi-C contact constraints, or functions of our structure’s coordinates.

(13) $$L\left(s\right)\;=\;\underset{t\in T}{\sum }\frac{n}{2}\;-\;log\left(\sqrt{\frac{\sum {\left(H_{i}\left\{t\right\}\;-\;D_{i}\left\{t\right\}\right)}^{2}}{n}}\right)\;+\;\underset{t\notin T}{\sum }[w_{1}\left(t\right)[\frac{n}{2}\;-\;log(\sqrt{\frac{\sum {\left(a_{1}\left\{t\right\}\;-\;D_{i}\left\{t\right\}\right)}^{2}}{n}})]\;+\;w_{2}\left(t\right)[\frac{n}{2}\;-\;log(\sqrt{\frac{\sum {\left(a_{2}\left\{t\right\}\;-\;D_{i}\left\{t\right\}\right)}^{2}}{n}})]$$

Because the purpose of 4DMax is to represent structural changes in time as a continuous evolution, rather than provide individual snapshot images, it is important that motion between frames be smooth. However, because the likelihood function (13) is predicated on satisfaction of distance constraints, and there is nothing incumbent within Equation (13) to ensure constant orientation. For a cognitive model, imagine a 6-sided die: over time, the interval is compressed into a 2-dimensional coin with the 6 and 1 faces becoming the faces of the coin. Suppose we are looking down at this die from the ceiling. The die is the same underlying structure regardless of whether it has a 6 as the upward face or a 3 as the upward face; however, it will look different from our viewpoint because of the differing orientation. Now imagine looking at this die as it deforms to the coin but assume that the orientation of the coin at each time point is random. This will result in a choppy visual representation, even if the deformation is continuous. Similarly, because the likelihood function (13) ensures satisfaction of spatial restraints but does not guide orientation and the positions of each genomic bin are randomly initialized, the 4D models could rotate excessively according to a motion that is guided by randomness, not biological or computational insight. To help prevent this uninformative rotation, we included a penalizing term, distance loss.

(14) $$D_{move}\left(s\right)\;=\;\underset{t}{\sum }\underset{i}{\sum }\sqrt{\underset{c}{\sum }{\left(S_{i,c}\left\{t\right\}\;-\;S_{i,c}\left\{t+1\right\}\right)}^{2};\;c\;\in \;\left\{x,y,z\right\}}$$

Inclusion of (14) in our loss function penalizes unnecessary movement of bins between frames.

We optimized our structure’s coordinates by constructing a linear combination of our distance loss function and likelihood-loss function and incrementally adjusting coordinate position via gradient descent, yielding (15)

(15) $$S{\left\{t\right\}}_{i_{x,y,z}}:=\;S{\left\{t\right\}}_{i_{x,y,z}}+\lambda \;*\;\left[-\nabla_{i}L\left(s\right)\;+\;\left(\eta *\nabla_{i}D_{move}\left(s\right)\right)\right]$$

where η is a weighing constant and λ is the learning rate. Unless stated otherwise, we ran experiments with λ=0.0001 and η=1000. Hyperparameters were determined by performing a grid search over values for λ, η, as well as the hyperparameter for distance to contact frequency ratio, γ (Section 4.2, Equation (16)). The optimal hyperparameters were selected based on average SRC between contact maps and structures at the 6 time points on chromosome 19 of the iPSC dataset (Supplementary Material Figure S19).

### 4.2. Interpolation of Contacts

We interpolated contacts by first running 4DMax, excluding input Hi-C maps at time t of interest. From the 4D model, we extracted the predicted 3D structure at time t. Using this 3D model, we assumed an inverse relationship between spatial distance and contact frequency using an equation frequently seen in the literature [11,13,14].

(16) $$IF_{i,j}=d_{i,j}^{\gamma }$$

We used γ=0.6 because this value performed best in model construction for our hyperparameter tuning (Supplementary Material Figure S19).

### 4.3. AB Compartment Analysis

A/B compartments were identified in the manner outlined by [5]. We first calculated observed over expected (O/E) matrices for contact maps, where expected values were the mean contact frequency between bins of a given distance. From O/E matrices, we treated rows as vectors and obtained Pearson correlation matrices. From the correlation matrices, we performed principal component analysis (PCA). We assigned compartments to each bin based on the sign of its corresponding row’s PC1 value. Trajectories were obtained by performing PCA on AB compartment sign assignment vectors. Scatterplots were obtained by mapping PC1 values between two corresponding AB profiles (x,y coordinates).

### 4.4. TAD Identification

TADs were identified using the directionality index approach [8], as implemented by HiCtool [7]. This procedure begins with the identification of a statistic called the directionality index on each genomic bin, using Equation (17):

(17) $$DI\;=\;\frac{B-A}{\left|B-A\right|}(\frac{{\left(A-E\right)}^{2}}{E}+\frac{{\left(B-E\right)}^{2}}{E}),\;E=\frac{A+B}{2}$$

where A is the number of reads mapped from a 50 kb bin to the upstream 2 Mb, B is the number of reads mapped from the same 50 kb bin downstream 2 Mb, and $E=\frac{\left(A+B\right)}{2}$. The method then uses this DI as an observation of a hidden directionality bias determined by a hidden Markov model. When calculating TAD borders we used contact maps at 50 kb resolution. When comparing TAD profiles, TADs with borders found withing 3 bins (150 kb) of each other were identified as having overlapping borders.

### 4.5. Statistical Analysis

We used multiple metrics for evaluating similarity of contact matrices and distance vectors of 3D structures, including Disparity ($M^{2}$), Pearson correlation coefficient (PCC), and Spearman correlation coefficient (SRC). Comparison between 4D structures were based on the average correlation between 3D structures at each corresponding time point. Comparison is computed by first aligning the two structures around the origin and normalizing the matrix representation of their bin’s 3D position (A) such that $Trace\left(AA^{T}\right)=1$ using the SciPy [28] Procrustes method. After this normalization, we computed disparity using the equation

(18) $$M^{2}=\underset{i\in bins}{\sum }{\left(S_{i}^{1}\left\{t\right\}\;-\;S_{i}^{2}\left\{t\right\}\right)}^{2}$$

### 4.6. Notes on HiC Data

For all experiments using iPSC data, excluding Section 2.9, we used contact matrices of 50 kb resolution. For all experiments using the cardio myocyte data we used 500 kb resolution. In Section 2.9, we used both 50 k and 500 kb resolution contact maps. Regions for which there was no experimental Hi-C data in all contact map time points were excluded from comparisons. The read count for the Cardiomyocyte data ranged from 88 million to 272 million reads per time point. The read count for the iPSC dataset ranged from 0.78 billion to 1.28 billion.

## Data Availability

All Hi-C data were downloaded from the Gene Expression Omnibus (GEO). Cardiomyocyte data was found at accession number GSE106690, and induced pluripotency data was found at the accession number GSE96611. All codes used to run experiments are available at https://github.com/Max-Highsmith/4DMax. (Access date 9 September 2021).

## Supplementary Materials

Supplementary materials can be found at https://www.mdpi.com/article/10.3390/ijms22189785/s1.
