# Supplementary material for: Four-Dimensional Chromosome Structure Prediction
Source: Int J Mol Sci. 2021 Sep 10;22(18):9785. doi: 10.3390/ijms22189785 (PMC8465368; doi:10.3390/ijms22189785)
Supplement: Supplementary file 1 [file ijms-22-09785-s001.zip › ijms-1350834-supplementary.pdf]

Supplementary Figures

Figure S1 Simulated Structure positions. **3**
Figure S2 Synthetic Contact maps **4**
Figure S3 iPSC 4DMax agreement with 3DMax. **5**
Figure S4 Synthetic Hi-C from 4D Structures **6**
Figure S5 Heat Maps Comparing iPSC reconstruction to iPSC Real Data **11**
Figure S7 Heat Maps Comparing Cardio Reconstruction **13**
Figure S8 Interpolated Hi-C from iPSC 4D Structure **16**
Figure S9 Interpolated Hi-C from Cardiomyocyte 4D Structures **20**
Figure S10 iPSC Contact Map Similarity **21**
Figure S11 Reconstructed iPSC Trajectory Curves **23**
Figure S12 iPSC AB Interpolation Scatter Plots **24**
Figure S13 Pearson Mats **30**
Figure S14 4D Model Similarity of Interpolated iPSC and Full iPSC Models **31**
Figure S15 4D Model Similarity of Interpolated Cardio and Cull Cardio **32**
Figure S16 Run Time **34**
Figure S17 Cardio Schematic **34**
Figure S18 AB Vec Pearson Matrices **40**
Figure S19 Hyper Parameter Tuning **42**
Figure S20 Performance Comparison to Numerical Interpolation **44**

Videos **45**
Video S1 Collection 1 Synthetic Videos 45
Video S2 Collection 2 iPSC Videos 45
Video S3 Collection 3 Cardio Videos 45
Video S4 Collection 4 iPSC Missing Chros 45
Video S5 Collection 5 Cardio Missing Chros 45
Video S6 Collection 6 Changing Resolution 45
Video S7 Collection 7 Changing Granularity 45

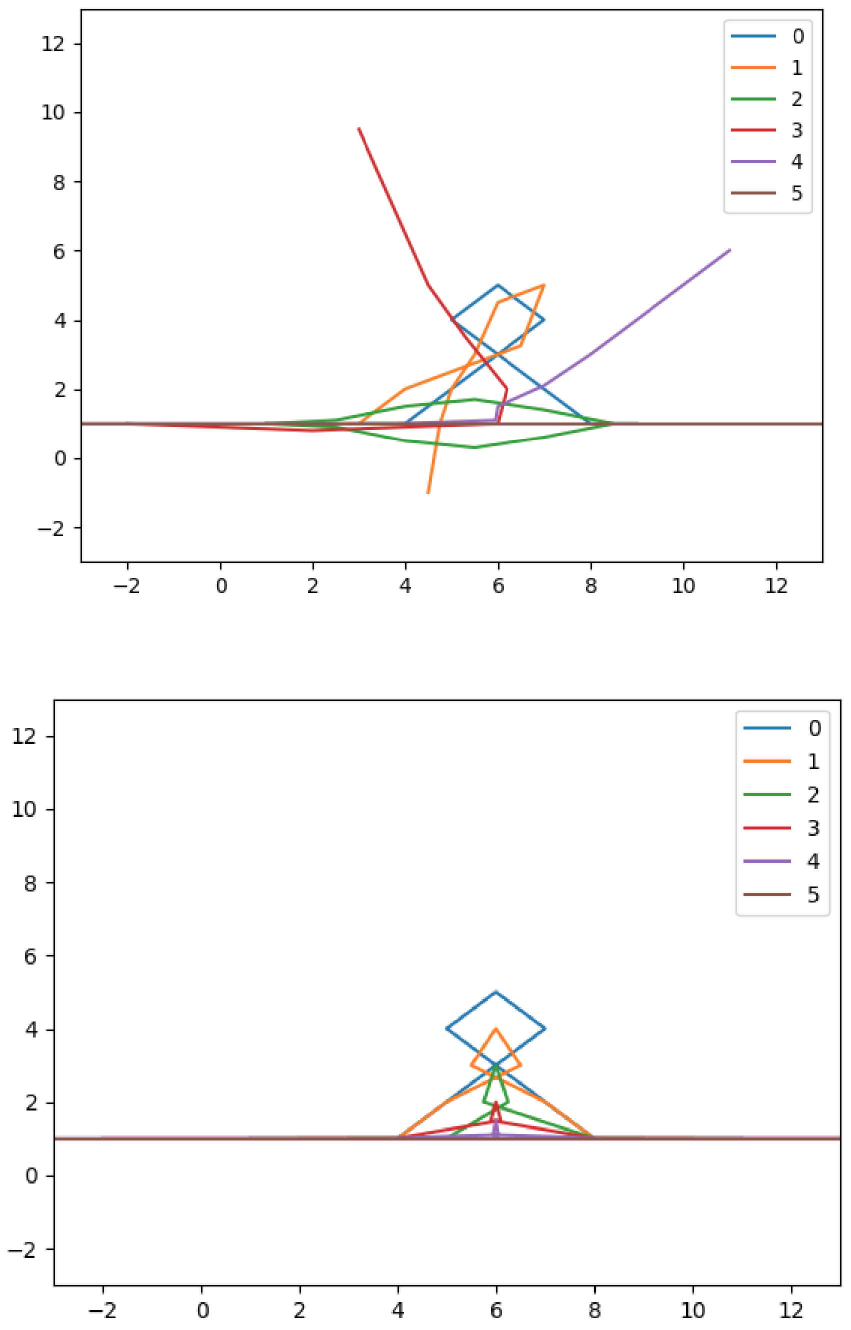

Figure S1 Simulated Structure positions.

Two dimensional projections of positions of synthetic chromosomes a. Structure 1. b. Structure 2. Each structure begins and ends with the same confirmation, but structure 1 is opened in a swinging motion while structure 2 is unraveled by both ends extending outward.

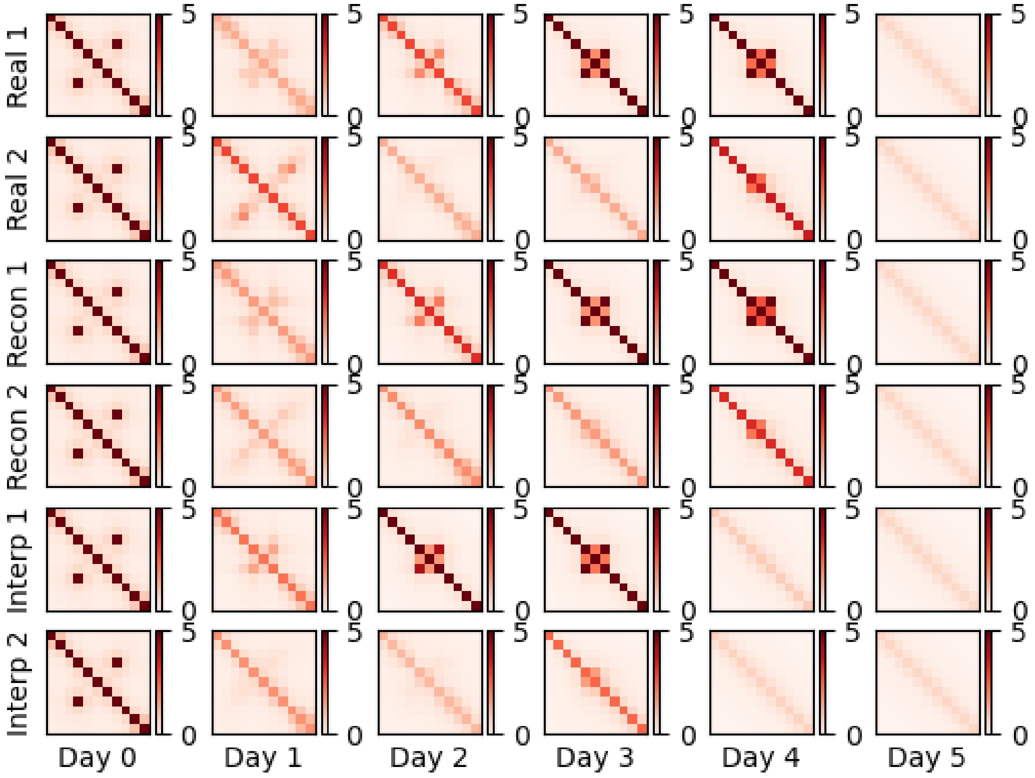

Figure S2 Synthetic Contact maps

Heatmaps of each day in each synthetic chromosome. The top 2 rows show heat maps of original synthetic data, middle two rows show heatmaps of 4DMax reconstructed structures and bottom two show interpolations if the algorithm 4DMax algorithm is not shown heat maps on given day.

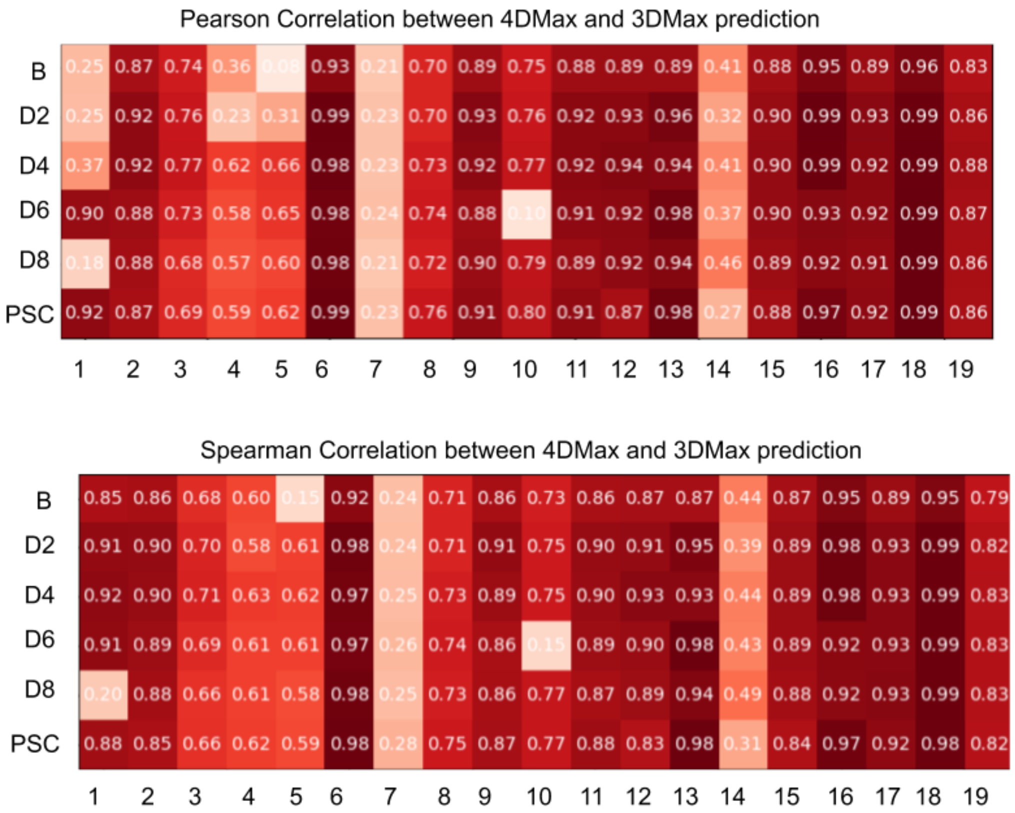

Figure S3 iPSC 4DMax agreement with 3DMax.

(a) pearson and (b) spearman correlation between 4DMax structures and 3D structure predicted using 3DMax algorithm.

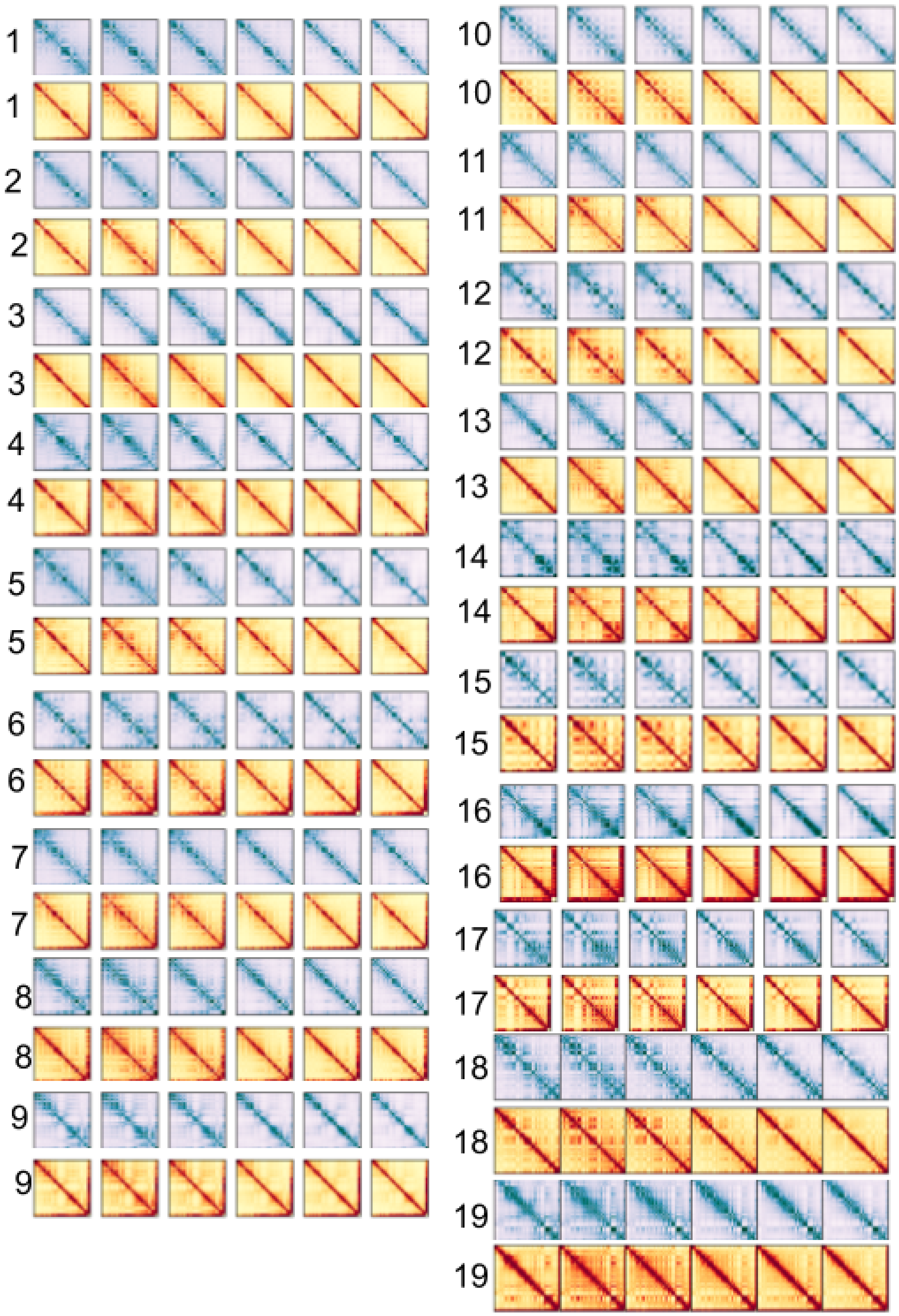

Figure S4 Synthetic Hi-C from 4D Structures

Chromosomal contact maps on each day for each chromosome in iPSC

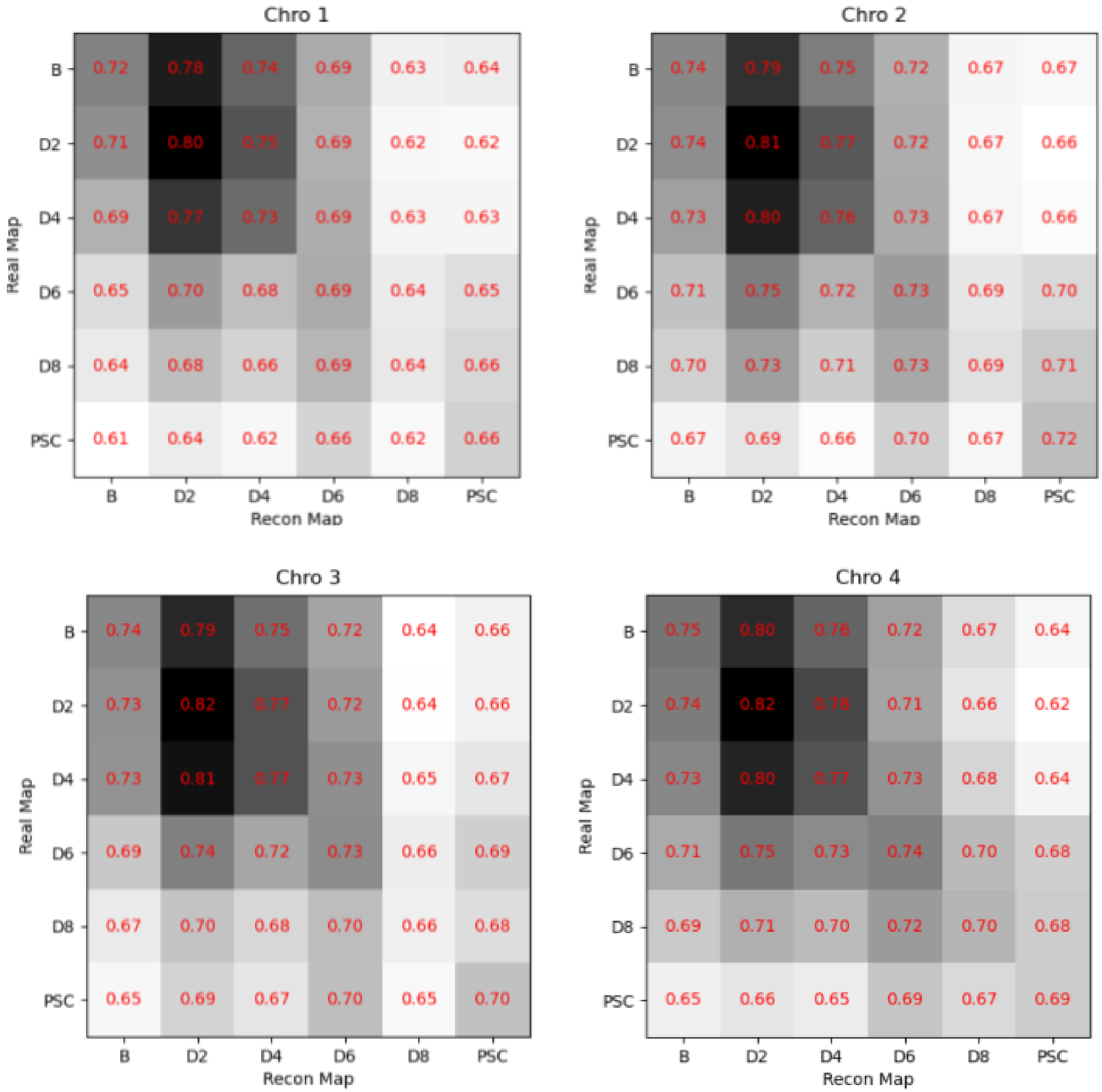

Chro 1
B 0.72 0.78 0.74 0.69 0.63 0.64
D2 0.71 0.80 0.75 0.69 0.62 0.62
D4 0.69 0.77 0.73 0.69 0.63 0.63
D6 0.65 0.70 0.68 0.69 0.64 0.65
D8 0.64 0.68 0.66 0.69 0.64 0.66
PSC 0.61 0.64 0.62 0.66 0.62 0.66
B D2 D4 D6 D8 PSC
Recon Map
Real Map
Chro 2
B 0.74 0.79 0.75 0.72 0.67 0.67
D2 0.74 0.81 0.77 0.72 0.67 0.66
D4 0.73 0.80 0.76 0.73 0.67 0.66
D6 0.71 0.75 0.72 0.73 0.69 0.70
D8 0.70 0.73 0.71 0.73 0.69 0.71
PSC 0.67 0.69 0.66 0.70 0.67 0.72
B D2 D4 D6 D8 PSC
Recon Map
Real Map
Chro 3
B 0.74 0.79 0.75 0.72 0.64 0.66
D2 0.73 0.82 0.77 0.72 0.64 0.66
D4 0.73 0.81 0.77 0.73 0.65 0.67
D6 0.69 0.74 0.72 0.73 0.66 0.69
D8 0.67 0.70 0.68 0.70 0.66 0.68
PSC 0.65 0.69 0.67 0.70 0.65 0.70
B D2 D4 D6 D8 PSC
Recon Map
Real Map
Chro 4
B 0.75 0.80 0.76 0.72 0.67 0.64
D2 0.74 0.82 0.78 0.71 0.66 0.62
D4 0.73 0.80 0.77 0.73 0.68 0.64
D6 0.71 0.75 0.73 0.74 0.70 0.68
D8 0.69 0.71 0.70 0.72 0.70 0.68
PSC 0.65 0.66 0.65 0.69 0.67 0.69
B D2 D4 D6 D8 PSC
Recon Map
Real Map

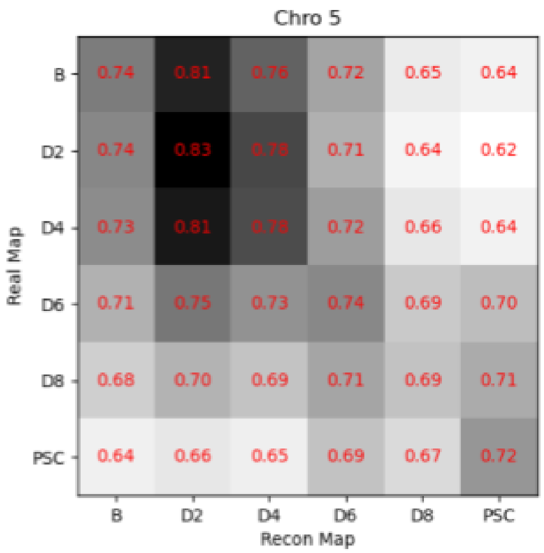
Chro 5
Real Map
Recon Map
B D2 D4 D6 D8 PSC
B 0.74 0.81 0.76 0.72 0.65 0.64
D2 0.74 0.83 0.78 0.71 0.64 0.62
D4 0.73 0.81 0.78 0.72 0.66 0.64
D6 0.71 0.75 0.73 0.74 0.69 0.70
D8 0.68 0.70 0.69 0.71 0.69 0.71
PSC 0.64 0.66 0.65 0.69 0.67 0.72

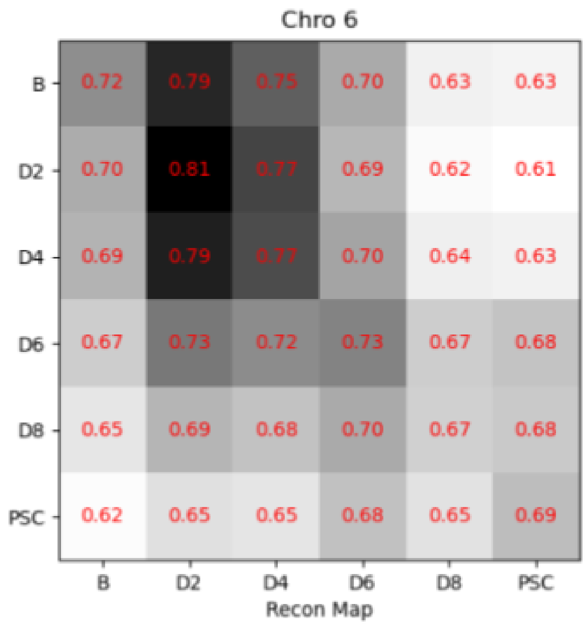
Chro 6
Recon Map
B D2 D4 D6 D8 PSC
B 0.72 0.79 0.75 0.70 0.63 0.63
D2 0.70 0.81 0.77 0.69 0.62 0.61
D4 0.69 0.79 0.77 0.70 0.64 0.63
D6 0.67 0.73 0.72 0.73 0.67 0.68
D8 0.65 0.69 0.68 0.70 0.67 0.68
PSC 0.62 0.65 0.65 0.68 0.65 0.69

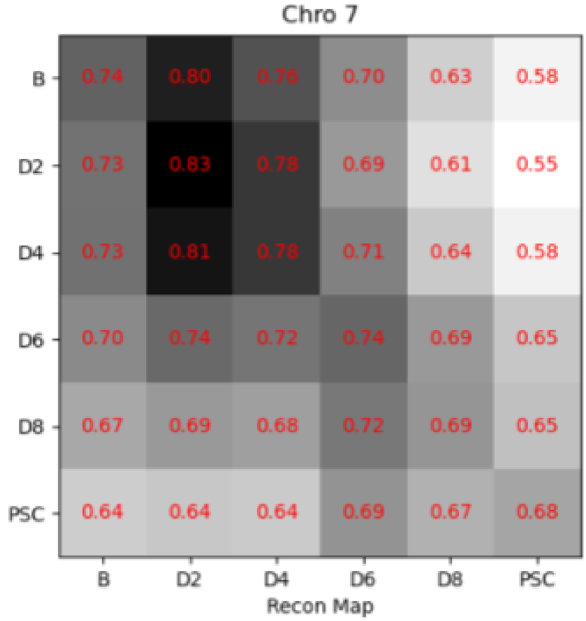
Chro 7
Recon Map
B D2 D4 D6 D8 PSC
B 0.74 0.80 0.76 0.70 0.63 0.58
D2 0.73 0.83 0.78 0.69 0.61 0.55
D4 0.73 0.81 0.78 0.71 0.64 0.58
D6 0.70 0.74 0.72 0.74 0.69 0.65
D8 0.67 0.69 0.68 0.72 0.69 0.65
PSC 0.64 0.64 0.64 0.69 0.67 0.68

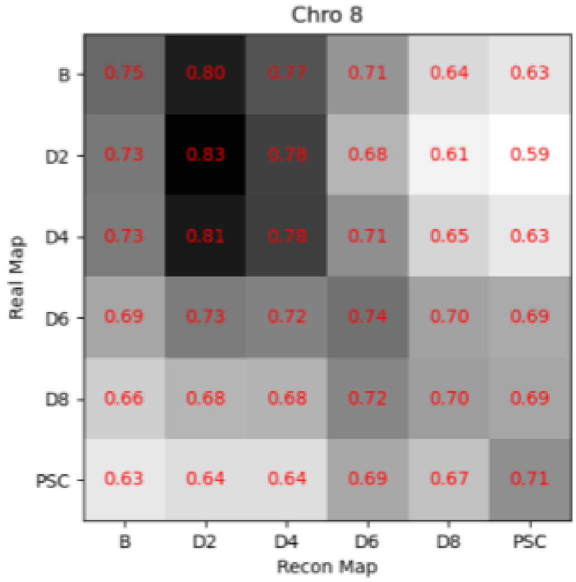
Chro 8
Real Map
Recon Map
B D2 D4 D6 D8 PSC
B 0.75 0.80 0.77 0.71 0.64 0.63
D2 0.73 0.83 0.78 0.68 0.61 0.59
D4 0.73 0.81 0.78 0.71 0.65 0.63
D6 0.69 0.73 0.72 0.74 0.70 0.69
D8 0.66 0.68 0.68 0.72 0.70 0.69
PSC 0.63 0.64 0.64 0.69 0.67 0.71

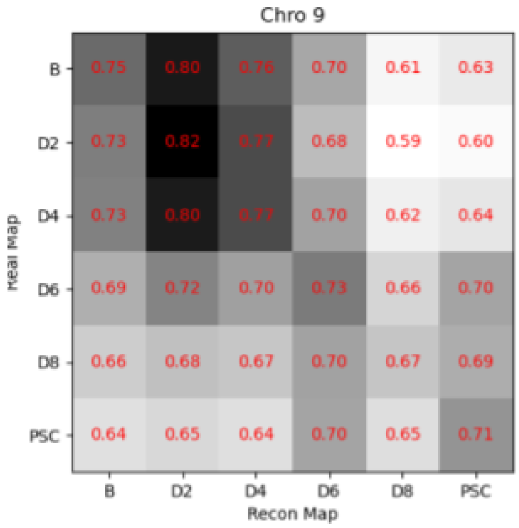
Chro 9
Real Map
Recon Map
B D2 D4 D6 D8 PSC
B 0.75 0.80 0.76 0.70 0.61 0.63
D2 0.73 0.82 0.77 0.68 0.59 0.60
D4 0.73 0.80 0.77 0.70 0.62 0.64
D6 0.69 0.72 0.70 0.73 0.66 0.70
D8 0.66 0.68 0.67 0.70 0.67 0.69
PSC 0.64 0.65 0.64 0.70 0.65 0.71

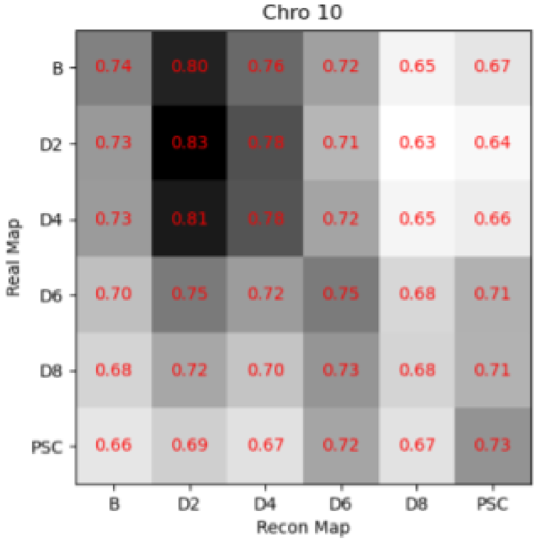
Chro 10
Real Map
Recon Map
B D2 D4 D6 D8 PSC
B 0.74 0.80 0.76 0.72 0.65 0.67
D2 0.73 0.83 0.78 0.71 0.63 0.64
D4 0.73 0.81 0.78 0.72 0.65 0.66
D6 0.70 0.75 0.72 0.75 0.68 0.71
D8 0.68 0.72 0.70 0.73 0.68 0.71
PSC 0.66 0.69 0.67 0.72 0.67 0.73

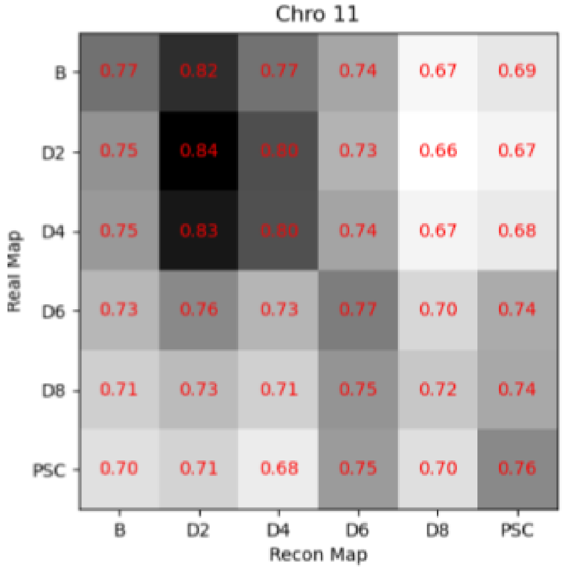
Chro 11
Real Map
Recon Map
B D2 D4 D6 D8 PSC
B 0.77 0.82 0.77 0.74 0.67 0.69
D2 0.75 0.84 0.80 0.73 0.66 0.67
D4 0.75 0.83 0.80 0.74 0.67 0.68
D6 0.73 0.76 0.73 0.77 0.70 0.74
D8 0.71 0.73 0.71 0.75 0.72 0.74
PSC 0.70 0.71 0.68 0.75 0.70 0.76

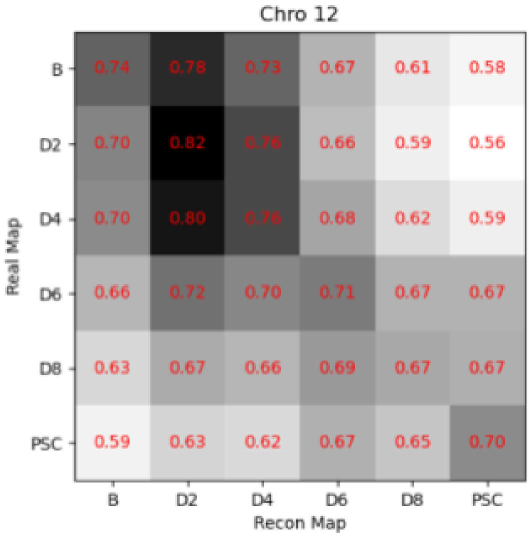
Chro 12
Real Map
Recon Map
B D2 D4 D6 D8 PSC
B 0.74 0.78 0.73 0.67 0.61 0.58
D2 0.70 0.82 0.76 0.66 0.59 0.56
D4 0.70 0.80 0.76 0.68 0.62 0.59
D6 0.66 0.72 0.70 0.71 0.67 0.67
D8 0.63 0.67 0.66 0.69 0.67 0.67
PSC 0.59 0.63 0.62 0.67 0.65 0.70

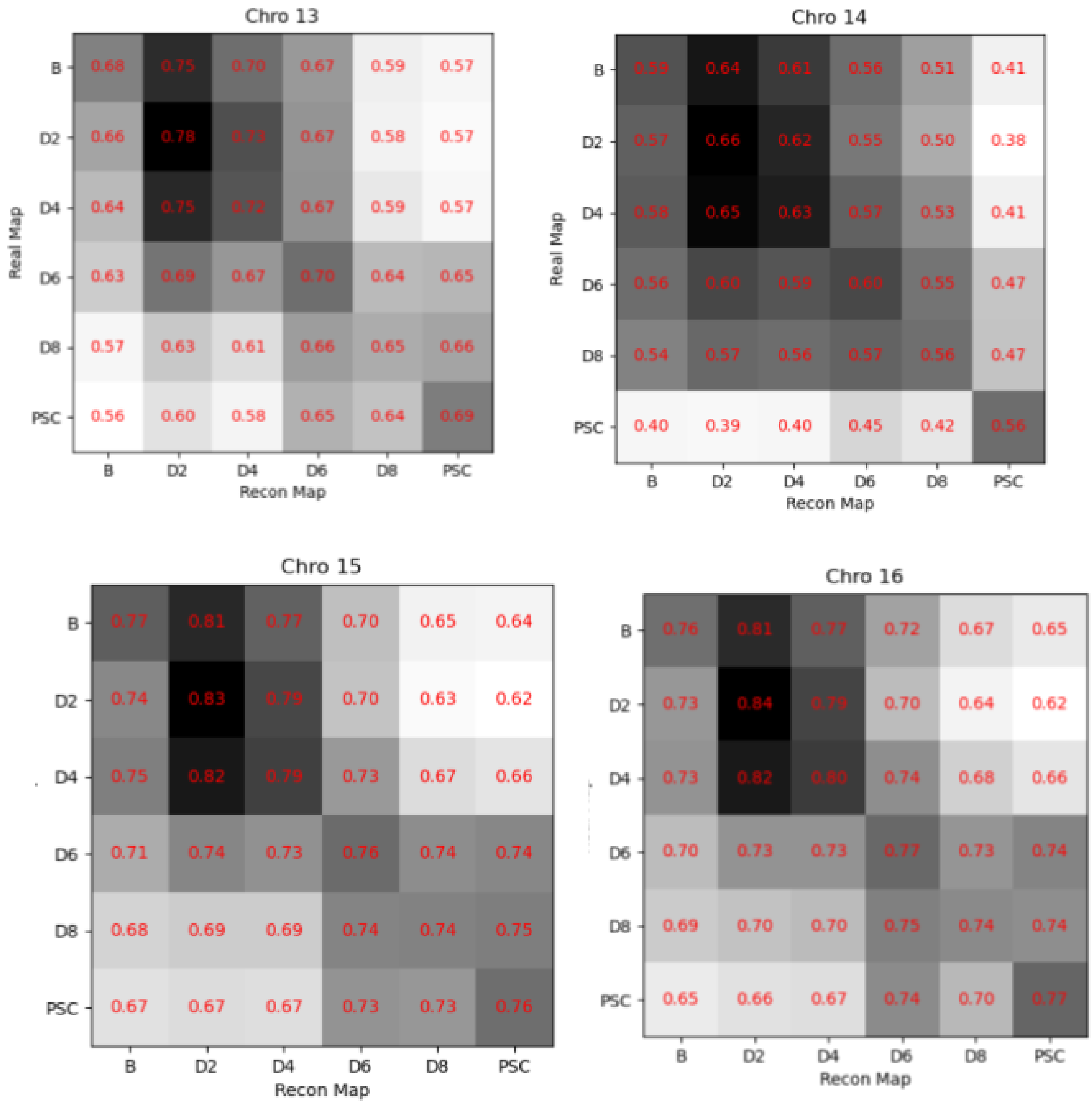
Chro 13
Real Map
Recon Map
B D2 D4 D6 D8 PSC
B 0.68 0.75 0.70 0.67 0.59 0.57
D2 0.66 0.78 0.73 0.67 0.58 0.57
D4 0.64 0.75 0.72 0.67 0.59 0.57
D6 0.63 0.69 0.67 0.70 0.64 0.65
D8 0.57 0.63 0.61 0.66 0.65 0.66
PSC 0.56 0.60 0.58 0.65 0.64 0.69
Chro 14
Real Map
Recon Map
B D2 D4 D6 D8 PSC
B 0.59 0.64 0.61 0.56 0.51 0.41
D2 0.57 0.66 0.62 0.55 0.50 0.38
D4 0.58 0.65 0.63 0.57 0.53 0.41
D6 0.56 0.60 0.59 0.60 0.55 0.47
D8 0.54 0.57 0.56 0.57 0.56 0.47
PSC 0.40 0.39 0.40 0.45 0.42 0.56
Chro 15
Recon Map
B D2 D4 D6 D8 PSC
B 0.77 0.81 0.77 0.70 0.65 0.64
D2 0.74 0.83 0.79 0.70 0.63 0.62
D4 0.75 0.82 0.79 0.73 0.67 0.66
D6 0.71 0.74 0.73 0.76 0.74 0.74
D8 0.68 0.69 0.69 0.74 0.74 0.75
PSC 0.67 0.67 0.67 0.73 0.73 0.76
Chro 16
Recon Map
B D2 D4 D6 D8 PSC
B 0.76 0.81 0.77 0.72 0.67 0.65
D2 0.73 0.84 0.79 0.70 0.64 0.62
D4 0.73 0.82 0.80 0.74 0.68 0.66
D6 0.70 0.73 0.73 0.77 0.73 0.74
D8 0.69 0.70 0.70 0.75 0.74 0.74
PSC 0.65 0.66 0.67 0.74 0.70 0.77

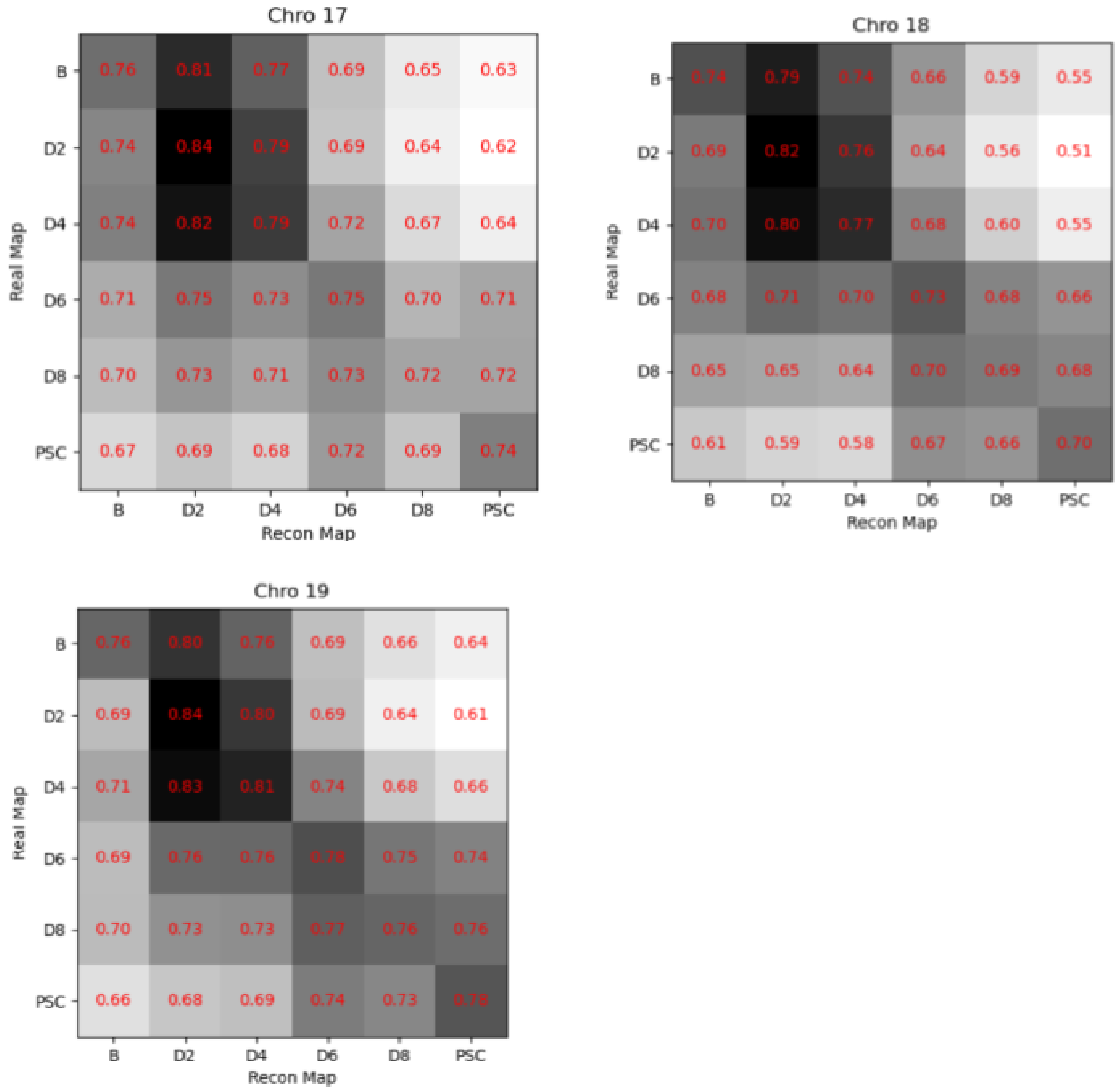

Figure S5 Heat Maps Comparing iPSC reconstruction to iPSC Real Data

Pearson correlation between real Hi-C contact maps and 4d reconstructed maps.

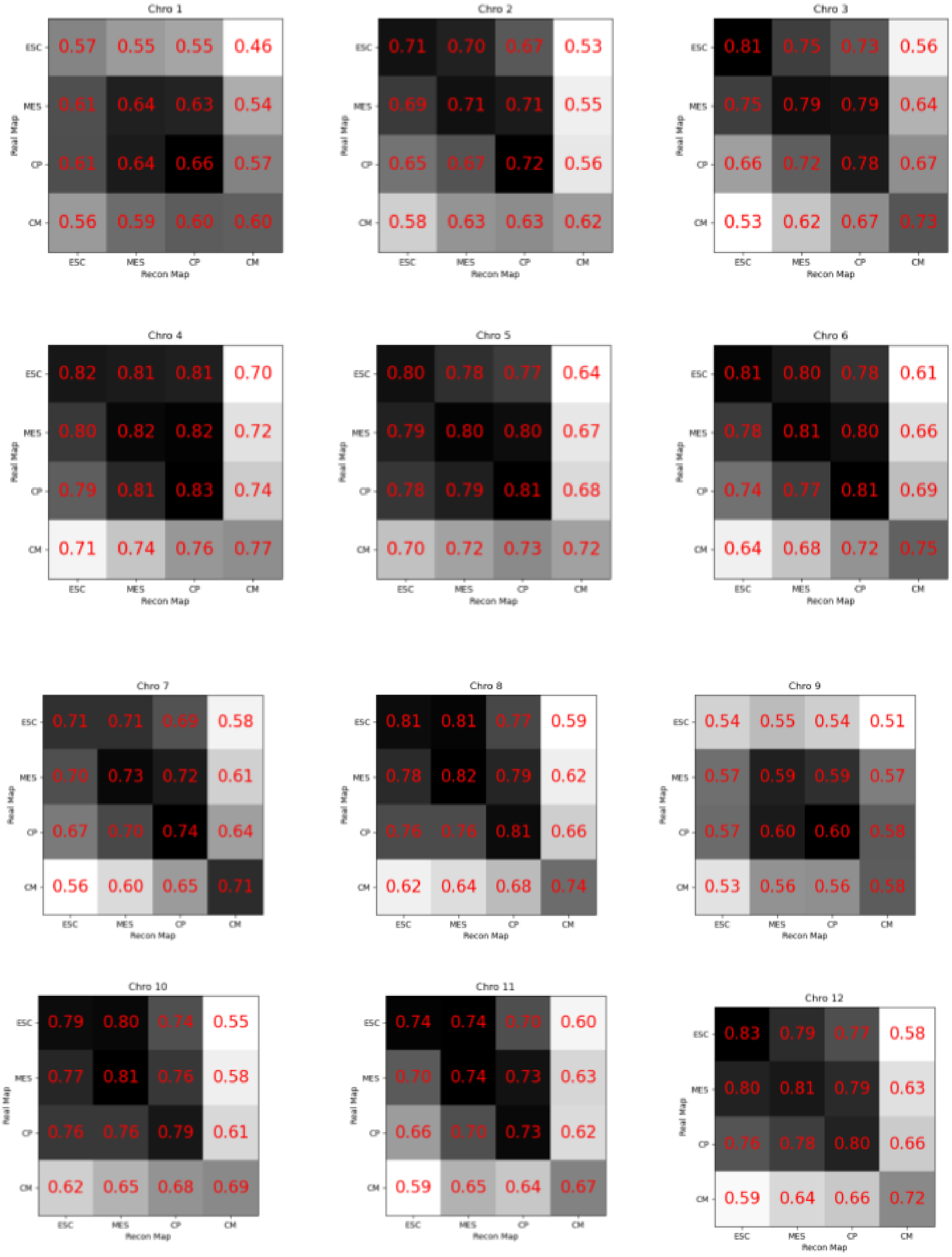
Chro 1
ESC 0.57 0.55 0.55 0.46
MES 0.61 0.64 0.63 0.54
CP 0.61 0.64 0.66 0.57
CM 0.56 0.59 0.60 0.60
Chro 2
ESC 0.71 0.70 0.67 0.53
MES 0.69 0.71 0.71 0.55
CP 0.65 0.67 0.72 0.56
CM 0.58 0.63 0.63 0.62
Chro 3
ESC 0.81 0.75 0.73 0.56
MES 0.75 0.79 0.79 0.64
CP 0.66 0.72 0.78 0.67
CM 0.53 0.62 0.67 0.73
Chro 4
ESC 0.82 0.81 0.81 0.70
MES 0.80 0.82 0.82 0.72
CP 0.79 0.81 0.83 0.74
CM 0.71 0.74 0.76 0.77
Chro 5
ESC 0.80 0.78 0.77 0.64
MES 0.79 0.80 0.80 0.67
CP 0.78 0.79 0.81 0.68
CM 0.70 0.72 0.73 0.72
Chro 6
ESC 0.81 0.80 0.78 0.61
MES 0.78 0.81 0.80 0.66
CP 0.74 0.77 0.81 0.69
CM 0.64 0.68 0.72 0.75
Chro 7
ESC 0.71 0.71 0.69 0.58
MES 0.70 0.73 0.72 0.61
CP 0.67 0.70 0.74 0.64
CM 0.56 0.60 0.65 0.71
Chro 8
ESC 0.81 0.81 0.77 0.59
MES 0.78 0.82 0.79 0.62
CP 0.76 0.76 0.81 0.66
CM 0.62 0.64 0.68 0.74
Chro 9
ESC 0.54 0.55 0.54 0.51
MES 0.57 0.59 0.59 0.57
CP 0.57 0.60 0.60 0.58
CM 0.53 0.56 0.56 0.58
Chro 10
ESC 0.79 0.80 0.74 0.55
MES 0.77 0.81 0.76 0.58
CP 0.76 0.76 0.79 0.61
CM 0.62 0.65 0.68 0.69
Chro 11
ESC 0.74 0.74 0.70 0.60
MES 0.70 0.74 0.73 0.63
CP 0.66 0.70 0.73 0.62
CM 0.59 0.65 0.64 0.67
Chro 12
ESC 0.83 0.79 0.77 0.58
MES 0.80 0.81 0.79 0.63
CP 0.76 0.78 0.80 0.66
CM 0.59 0.64 0.66 0.72
ESC MES CP CM
Recon Map
Real Map

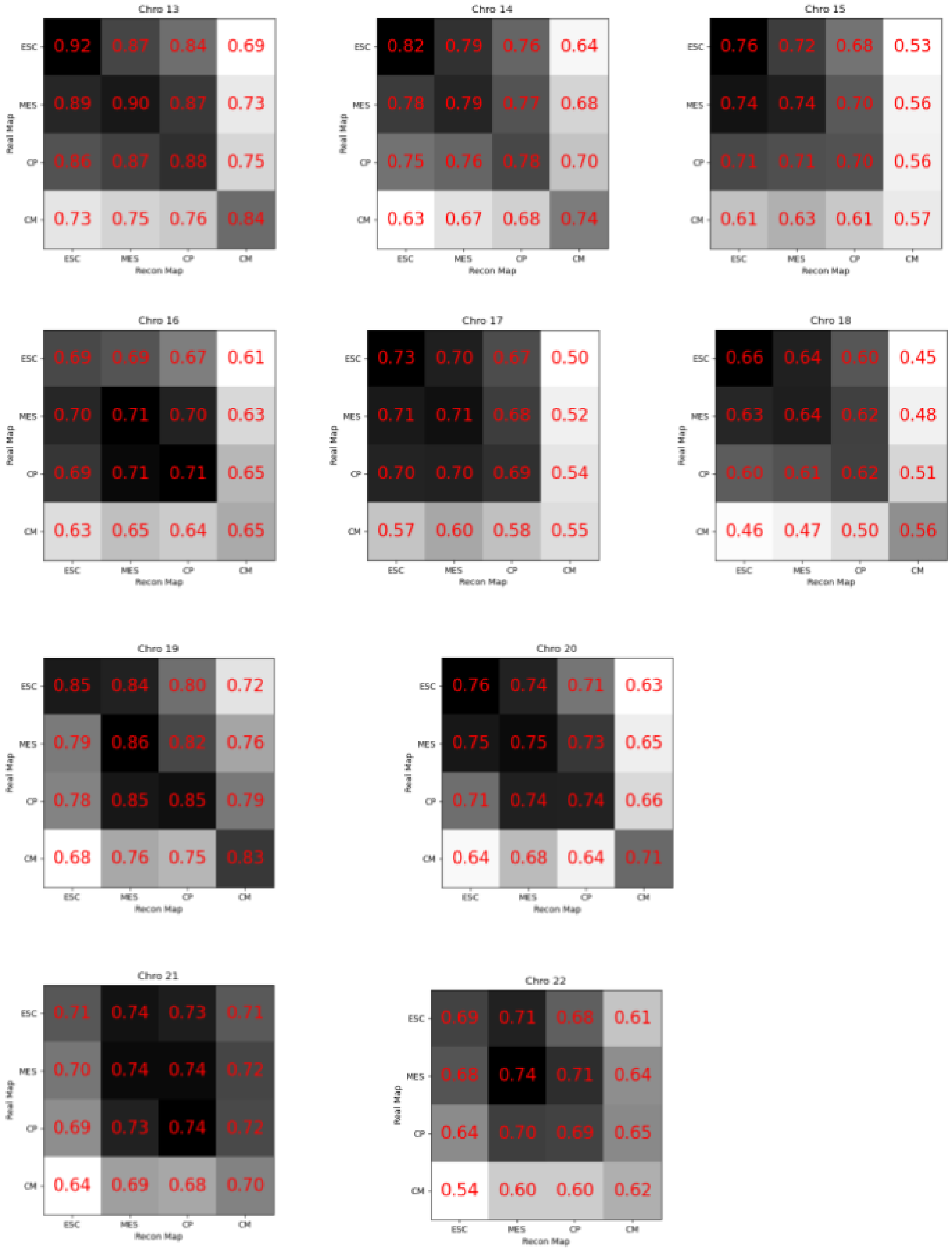

Figure S7 Heat Maps Comparing Cardio Reconstruction

Pearson correlation between real Hi-C contact maps and 4d reconstructed maps.

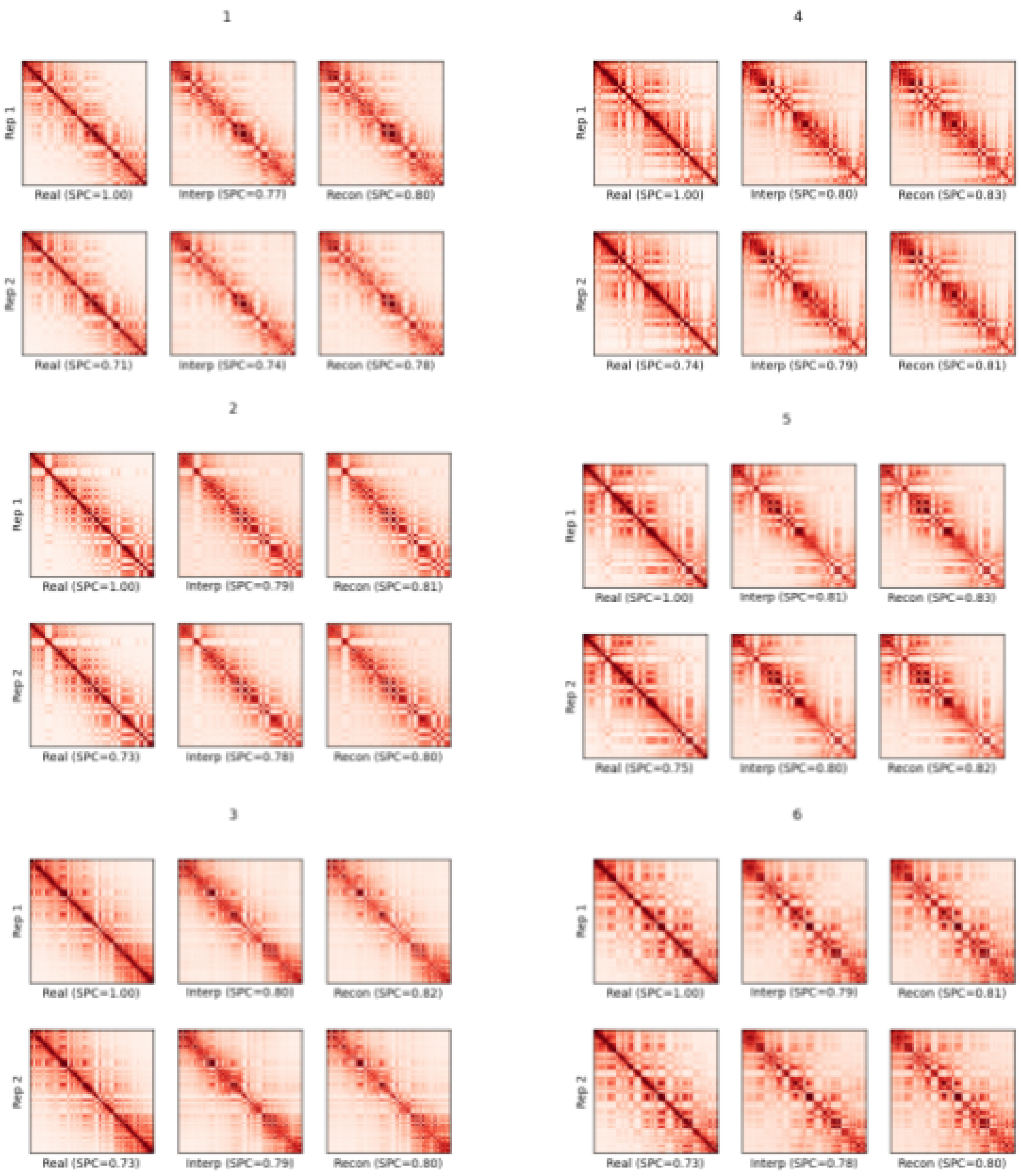

1
Rep 1
Real (SPC=1.00)
Interp (SPC=0.77)
Recon (SPC=0.80)
Rep 2
Real (SPC=0.71)
Interp (SPC=0.74)
Recon (SPC=0.78)
4
Rep 1
Real (SPC=1.00)
Interp (SPC=0.80)
Recon (SPC=0.83)
Rep 2
Real (SPC=0.74)
Interp (SPC=0.79)
Recon (SPC=0.81)
2
Rep 1
Real (SPC=1.00)
Interp (SPC=0.79)
Recon (SPC=0.81)
Rep 2
Real (SPC=0.73)
Interp (SPC=0.78)
Recon (SPC=0.80)
5
Rep 1
Real (SPC=1.00)
Interp (SPC=0.81)
Recon (SPC=0.83)
Rep 2
Real (SPC=0.75)
Interp (SPC=0.80)
Recon (SPC=0.82)
3
Rep 1
Real (SPC=1.00)
Interp (SPC=0.80)
Recon (SPC=0.82)
Rep 2
Real (SPC=0.73)
Interp (SPC=0.79)
Recon (SPC=0.80)
6
Rep 1
Real (SPC=1.00)
Interp (SPC=0.79)
Recon (SPC=0.81)
Rep 2
Real (SPC=0.73)
Interp (SPC=0.78)
Recon (SPC=0.80)

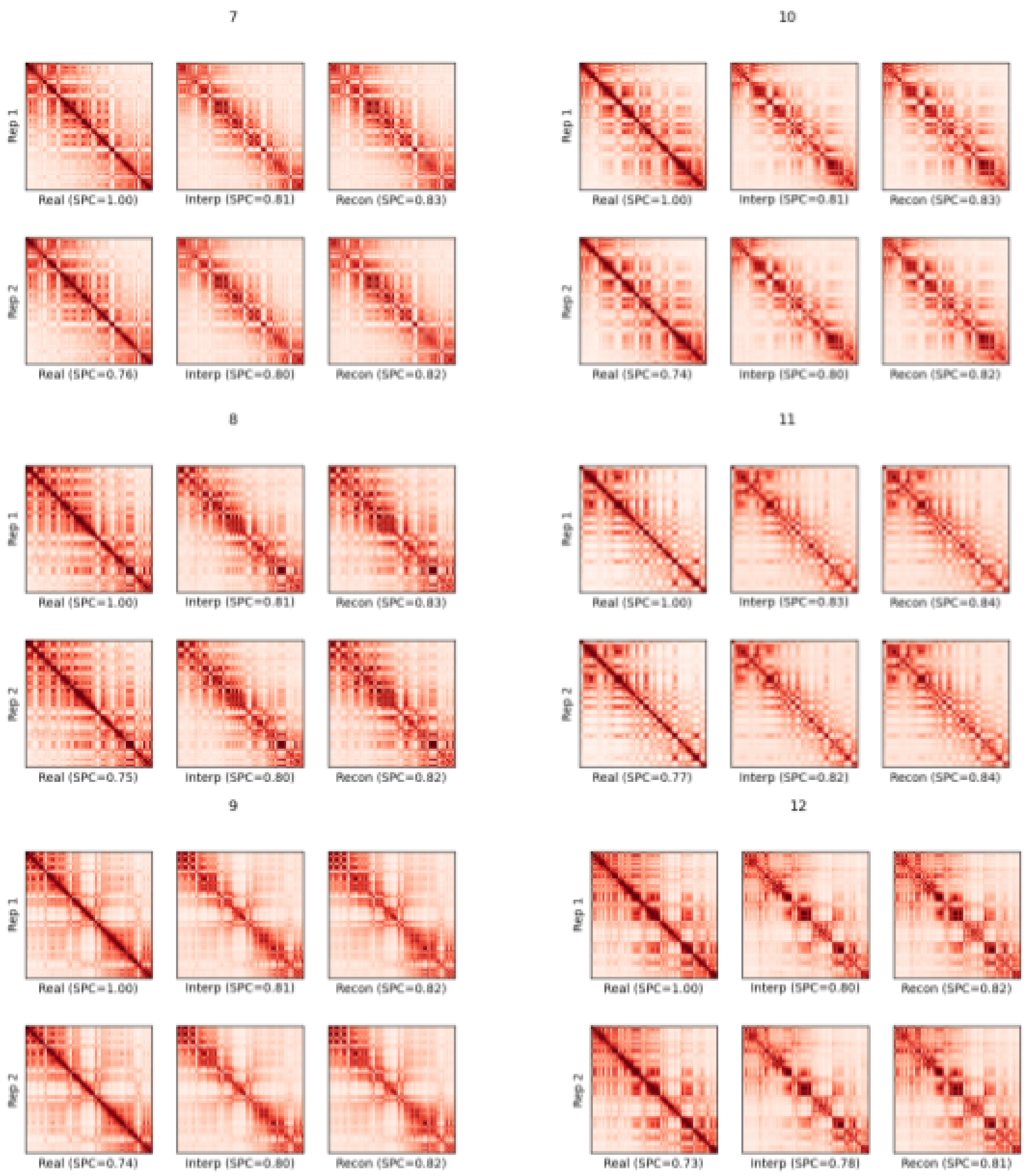

7
Rep 1
Real (SPC=1.00)
Interp (SPC=0.81)
Recon (SPC=0.83)
Rep 2
Real (SPC=0.76)
Interp (SPC=0.80)
Recon (SPC=0.82)
10
Rep 1
Real (SPC=1.00)
Interp (SPC=0.81)
Recon (SPC=0.83)
Rep 2
Real (SPC=0.74)
Interp (SPC=0.80)
Recon (SPC=0.82)
8
Rep 1
Real (SPC=1.00)
Interp (SPC=0.81)
Recon (SPC=0.83)
Rep 2
Real (SPC=0.75)
Interp (SPC=0.80)
Recon (SPC=0.82)
11
Rep 1
Real (SPC=1.00)
Interp (SPC=0.83)
Recon (SPC=0.84)
Rep 2
Real (SPC=0.77)
Interp (SPC=0.82)
Recon (SPC=0.84)
9
Rep 1
Real (SPC=1.00)
Interp (SPC=0.81)
Recon (SPC=0.82)
Rep 2
Real (SPC=0.74)
Interp (SPC=0.80)
Recon (SPC=0.82)
12
Rep 1
Real (SPC=1.00)
Interp (SPC=0.80)
Recon (SPC=0.82)
Rep 2
Real (SPC=0.73)
Interp (SPC=0.78)
Recon (SPC=0.81)

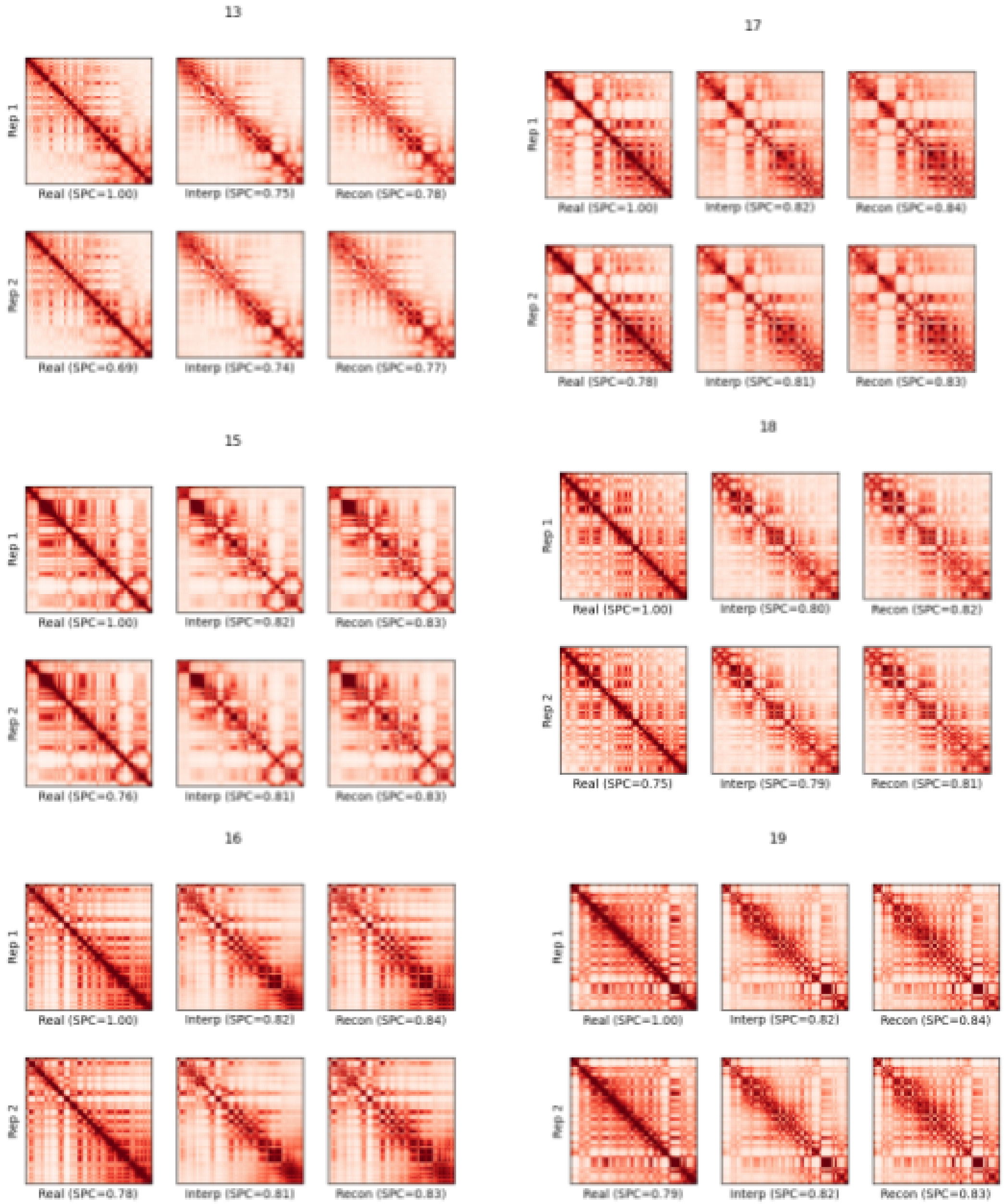

Figure S8 Interpolated Hi-C from iPSC 4D Structure

(left) Real Hi-C (middle) reconstructed Hi-C contact maps (right) interpolated contact maps.

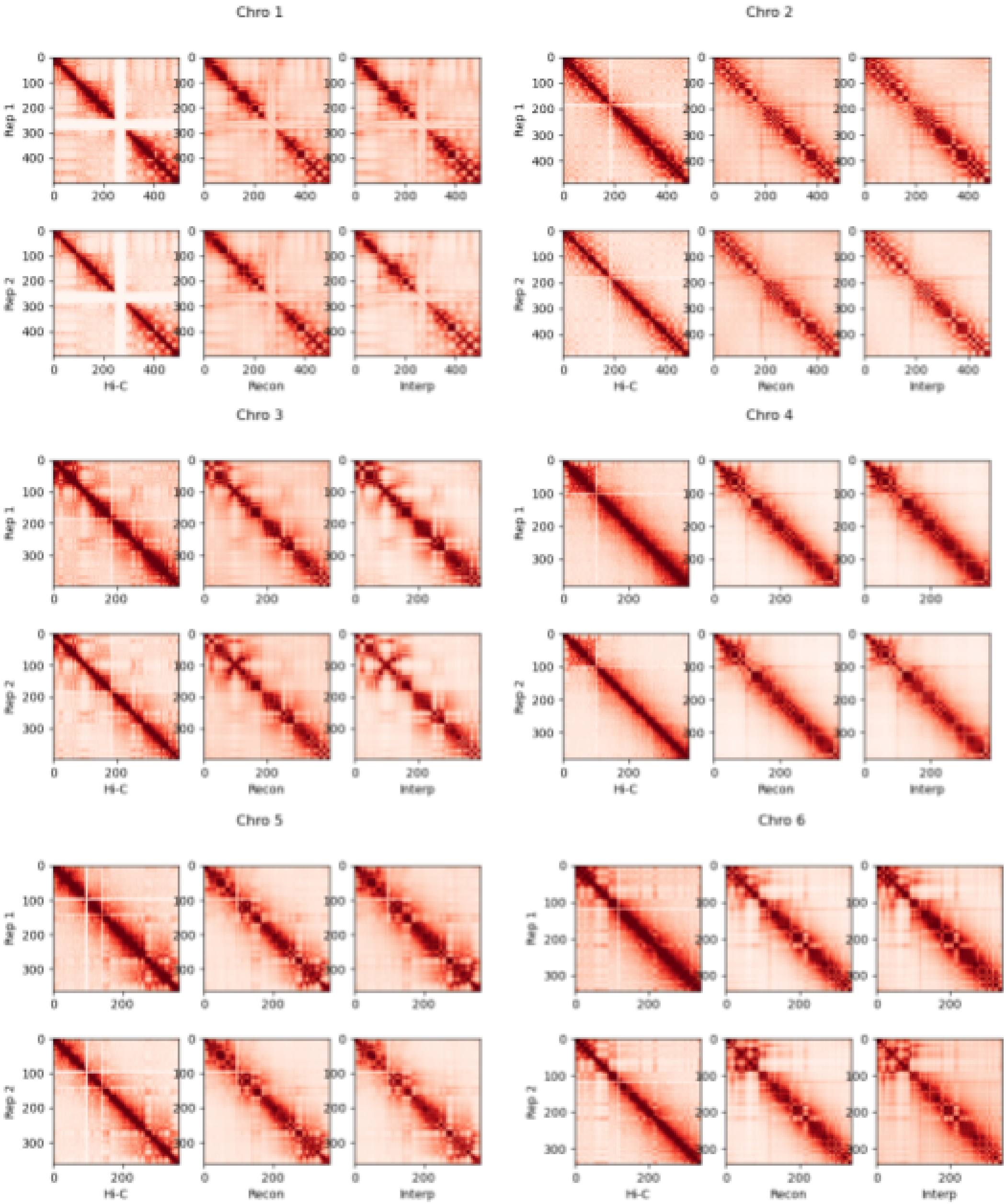

Chro 1
Chro 2
Chro 3
Chro 4
Chro 5
Chro 6
Rep 1
Rep 2
Hi-C
Recon
Interp

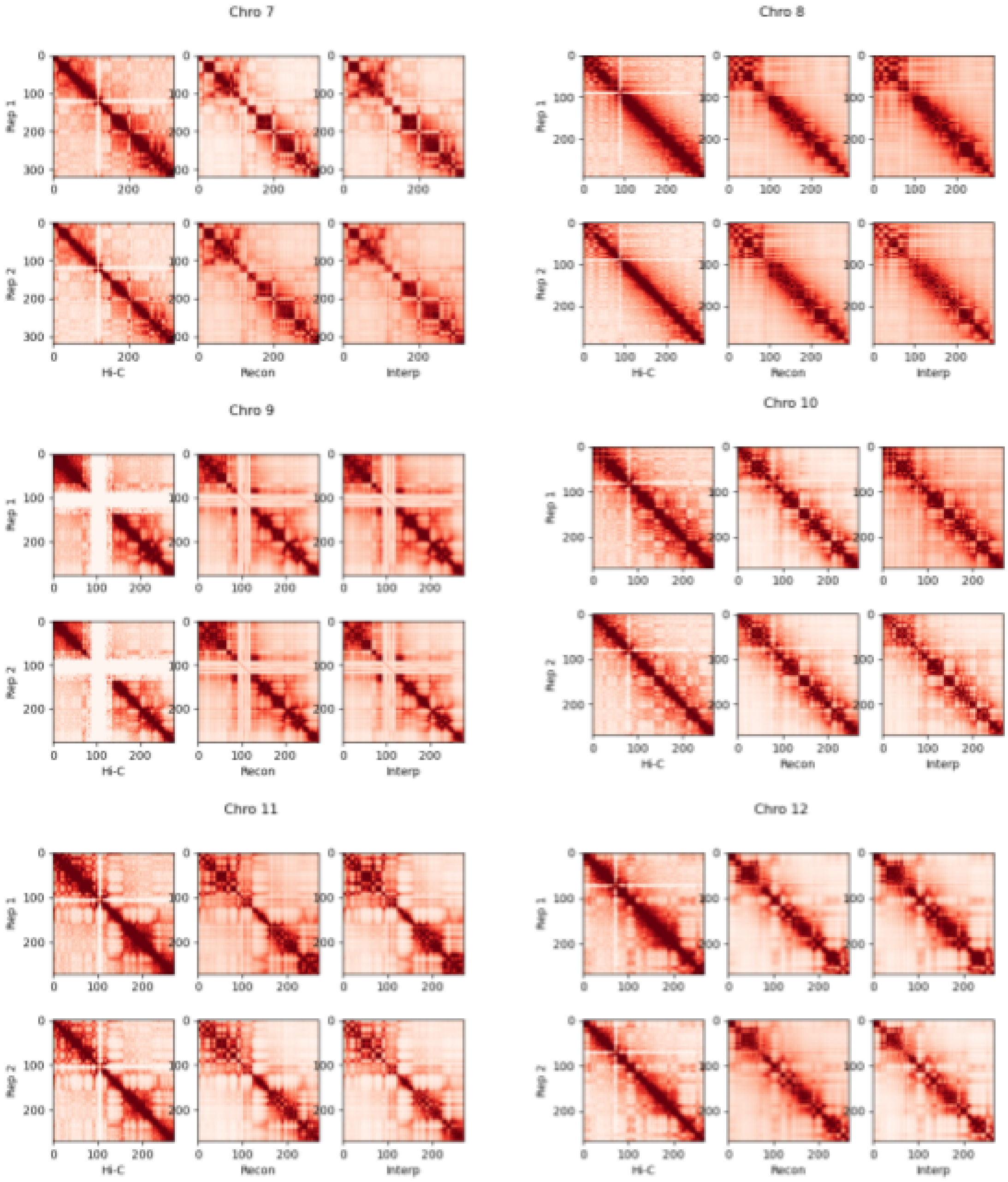
Chro 7
Chro 8
Chro 9
Chro 10
Chro 11
Chro 12
Rep 1
Rep 2
Hi-C
Recon
Interp

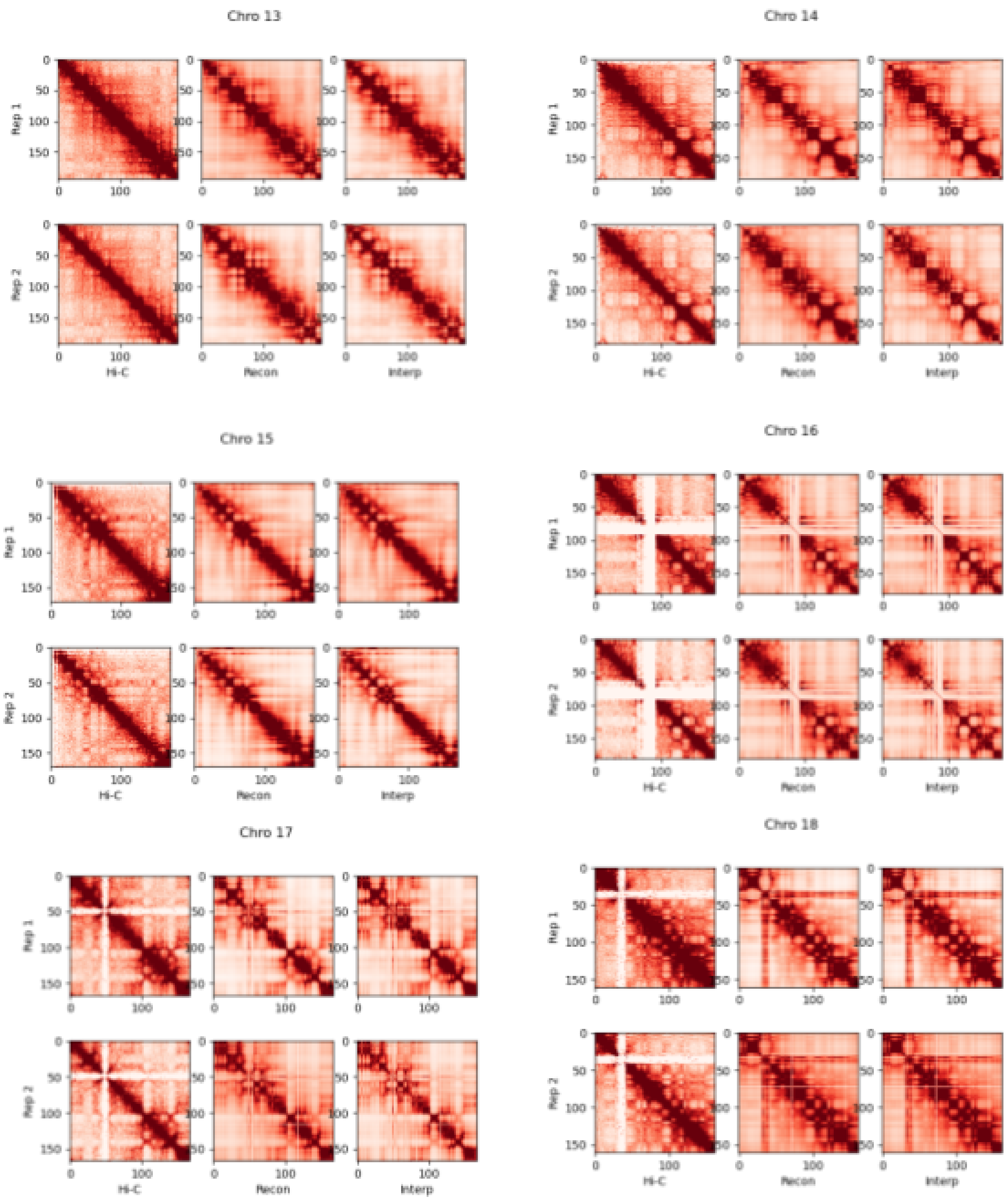

Chro 13
Chro 14
Chro 15
Chro 16
Chro 17
Chro 18
Rep 1
Rep 2
Hi-C
Recon
Interp

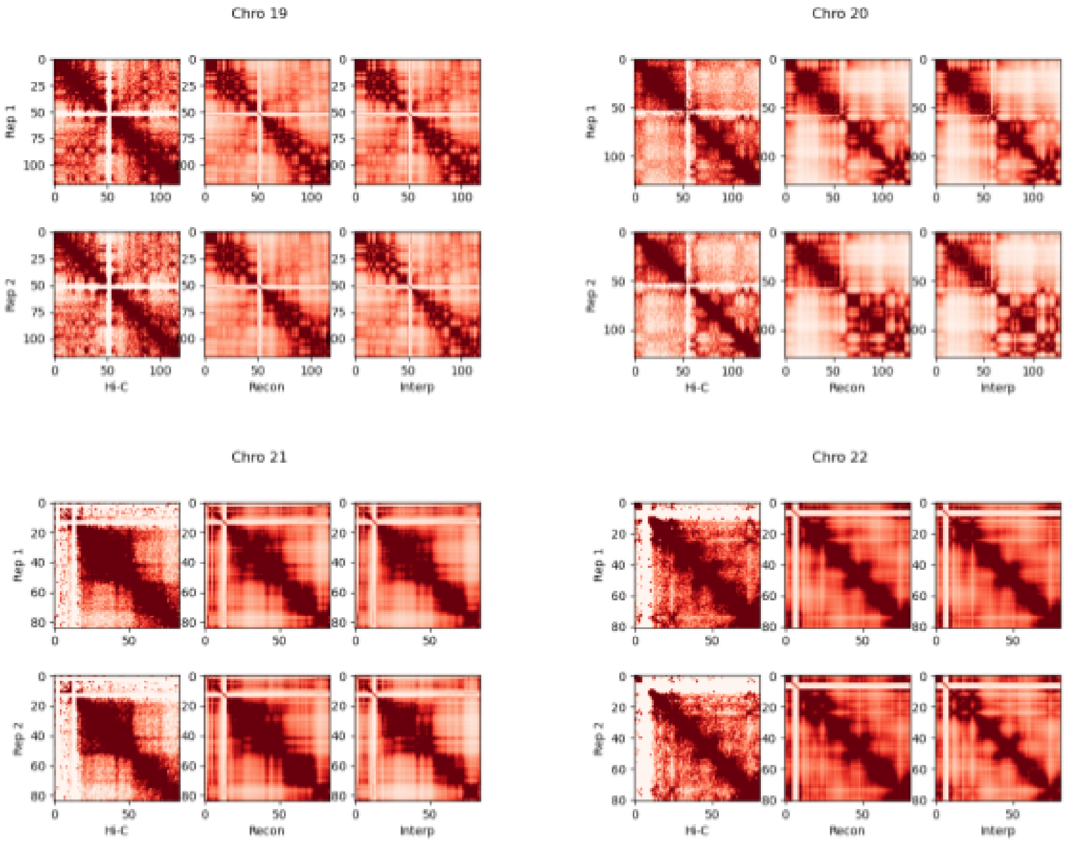

Figure S9 Interpolated Hi-C from Cardiomyocyte 4D Structures

(left) Real Hi-C (middle) reconstructed Hi-C contact maps (right) interpolated contact maps.

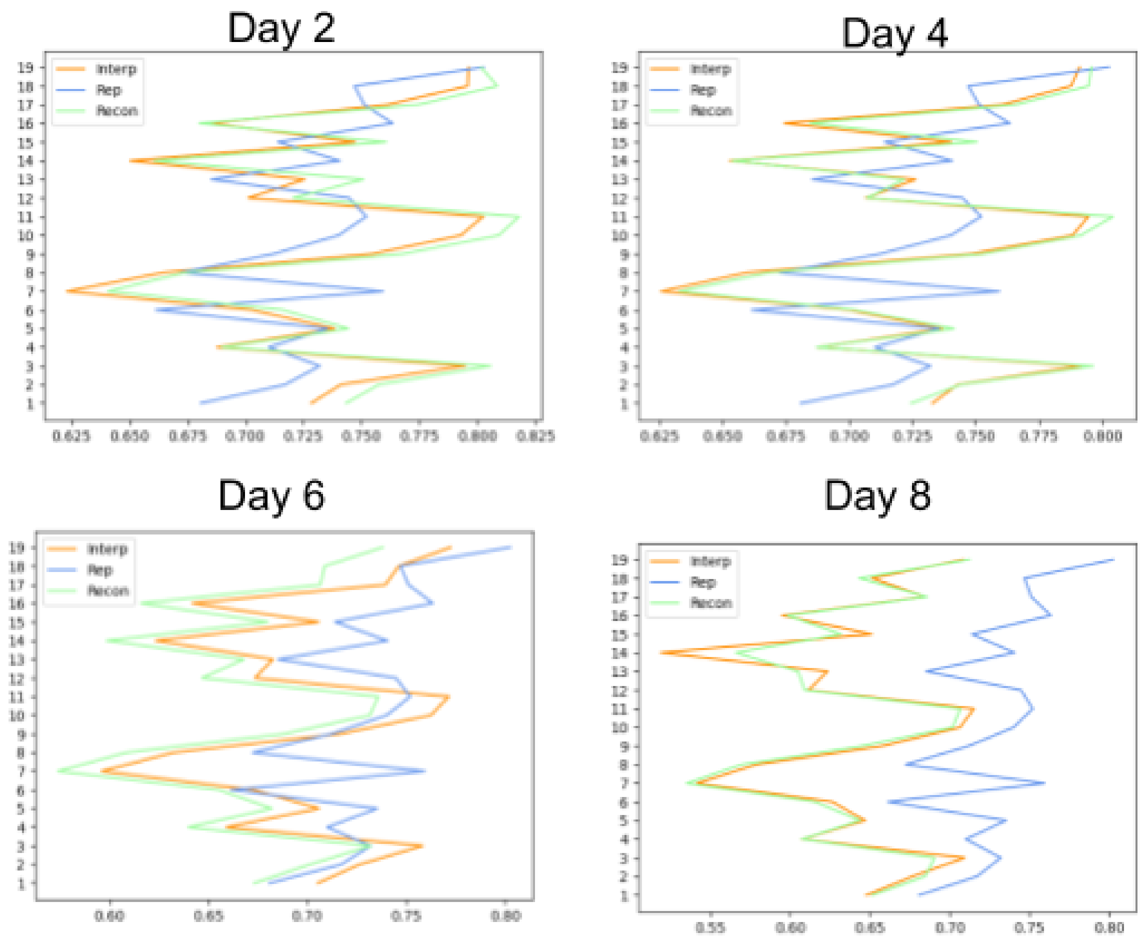

Figure S10 iPSC Contact Map Similarity

(orange) interpolation (green) reconstruction and (blue) biological replication.

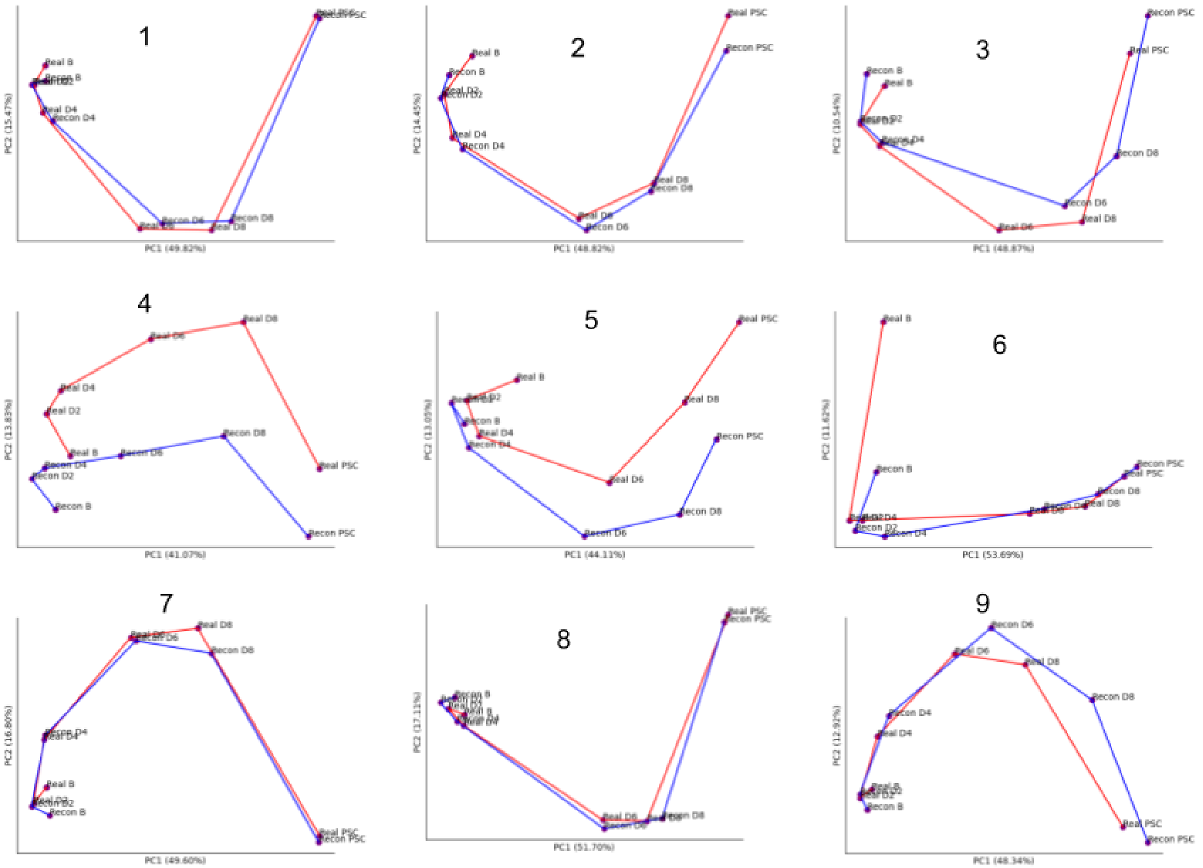

1
PC1 (49.82%)
PC2 (15.47%)
2
PC1 (48.82%)
PC2 (14.45%)
3
PC1 (48.87%)
PC2 (10.54%)
4
PC1 (41.07%)
PC2 (13.83%)
5
PC1 (44.11%)
PC2 (13.05%)
6
PC1 (53.69%)
PC2 (11.62%)
7
PC1 (49.60%)
PC2 (16.80%)
8
PC1 (51.70%)
PC2 (17.11%)
9
PC1 (48.34%)
PC2 (12.92%)

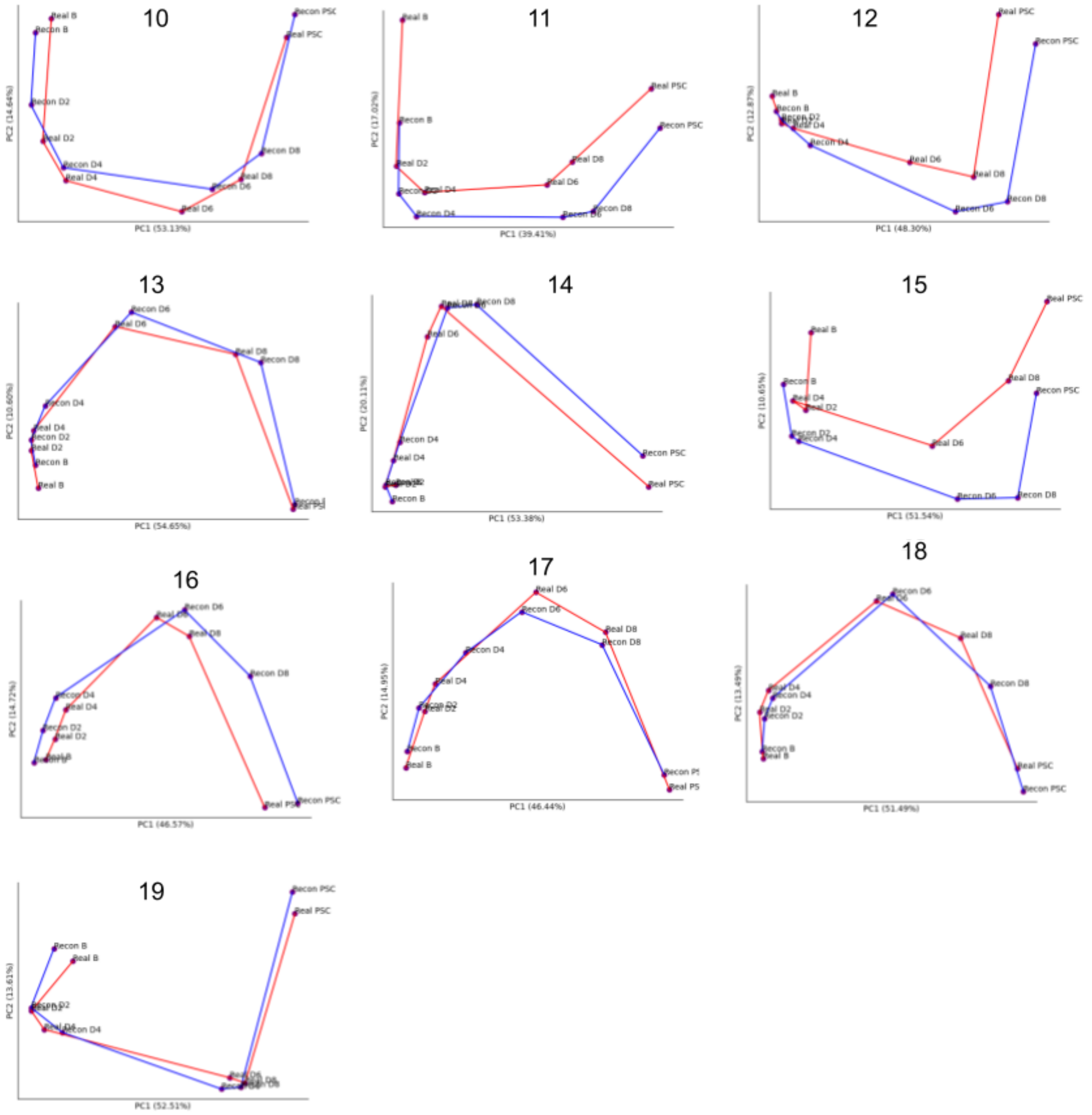

Figure S11 Reconstructed iPSC Trajectory Curves

(red) real Hi-C Trajectory (blue) Reconstructed Hi-C trajectory

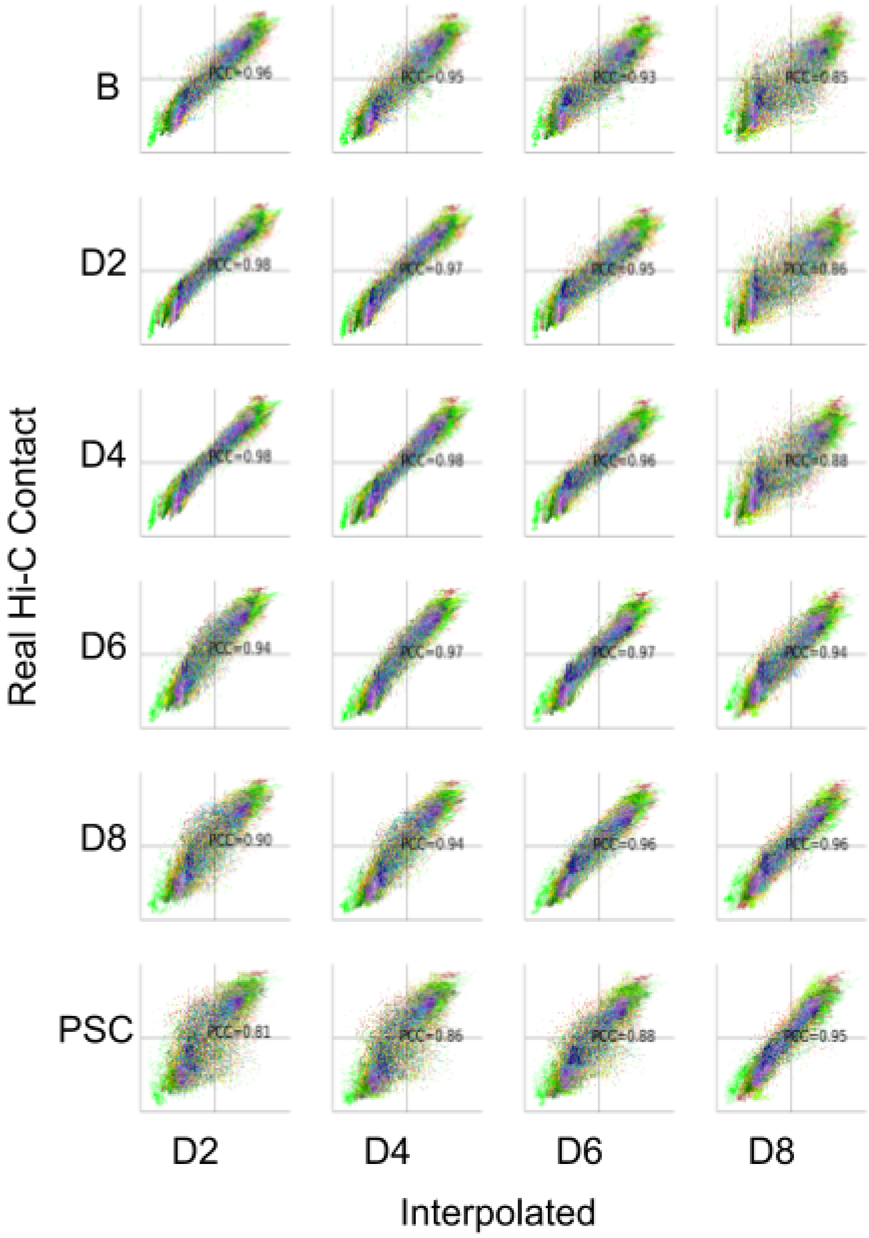

Figure S12 iPSC AB Interpolation Scatter Plots

Scatter plot of PC1 values from interpolated and biological real Hi-C contact maps.

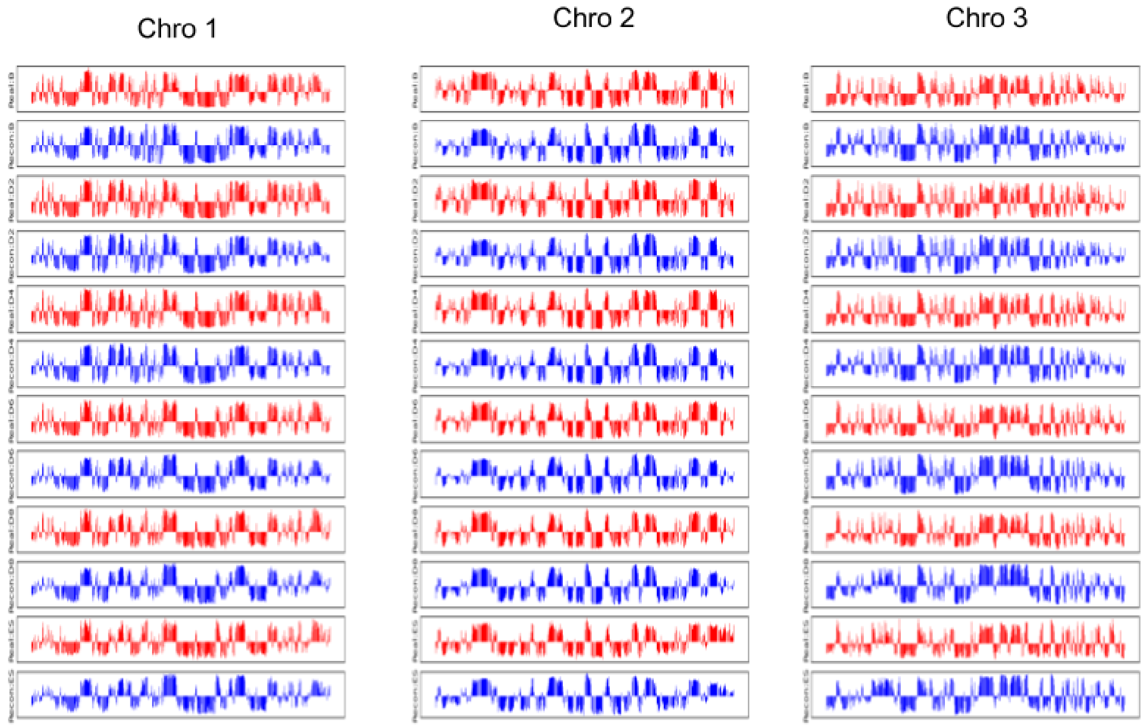
Chro 1
Chro 2
Chro 3

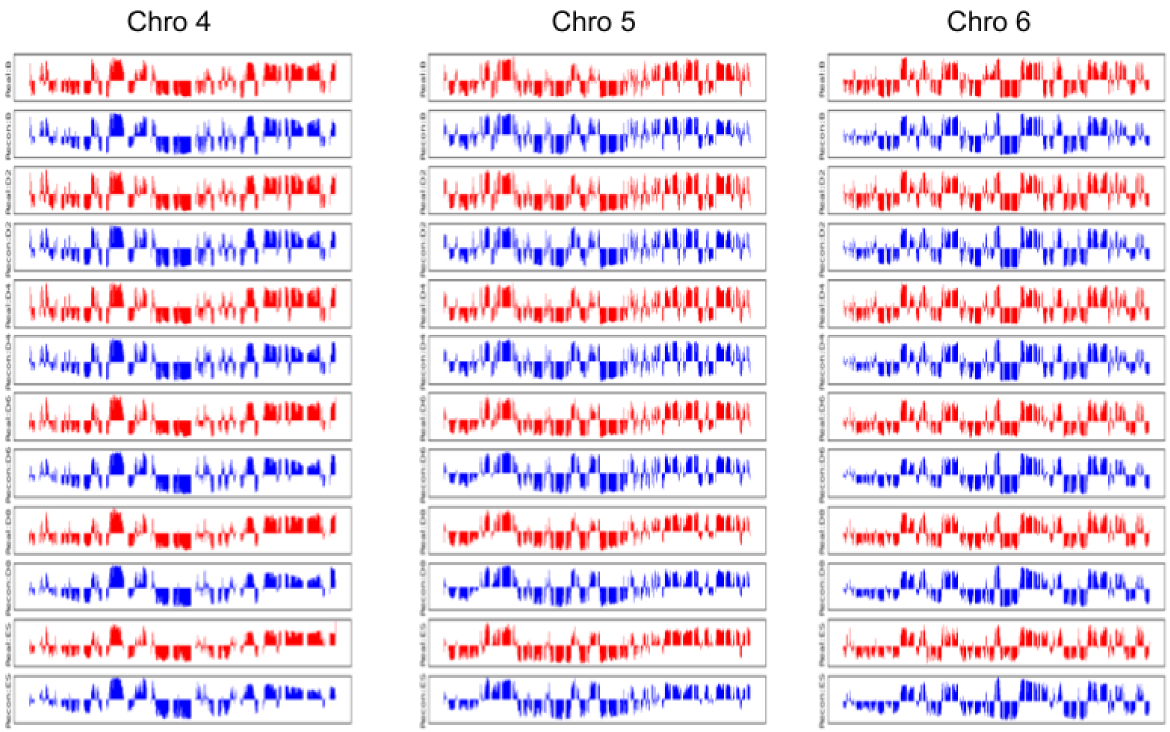
Chro 4
Chro 5
Chro 6
Real:B
Recon:B
Real:D2
Recon:D2
Real:D4
Recon:D4
Real:D6
Recon:D6
Real:D8
Recon:D8
Real:ES
Recon:ES

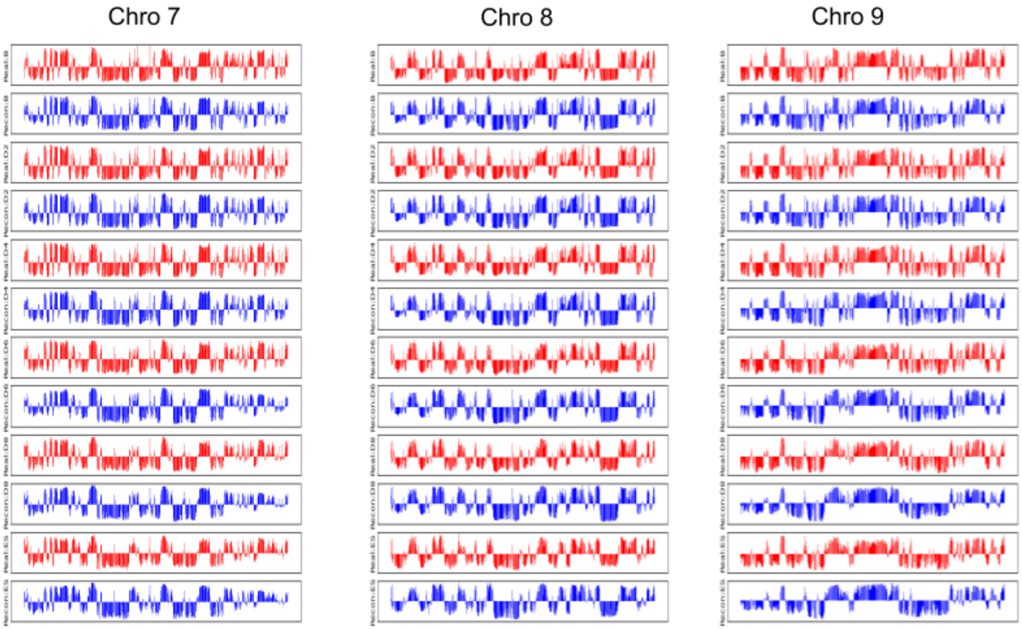
Chro 7
Chro 8
Chro 9
Real:D2
Recon:D2
Real:D4
Recon:D4
Real:D6
Recon:D6
Real:D8
Recon:D8
Real:ES
Recon:ES

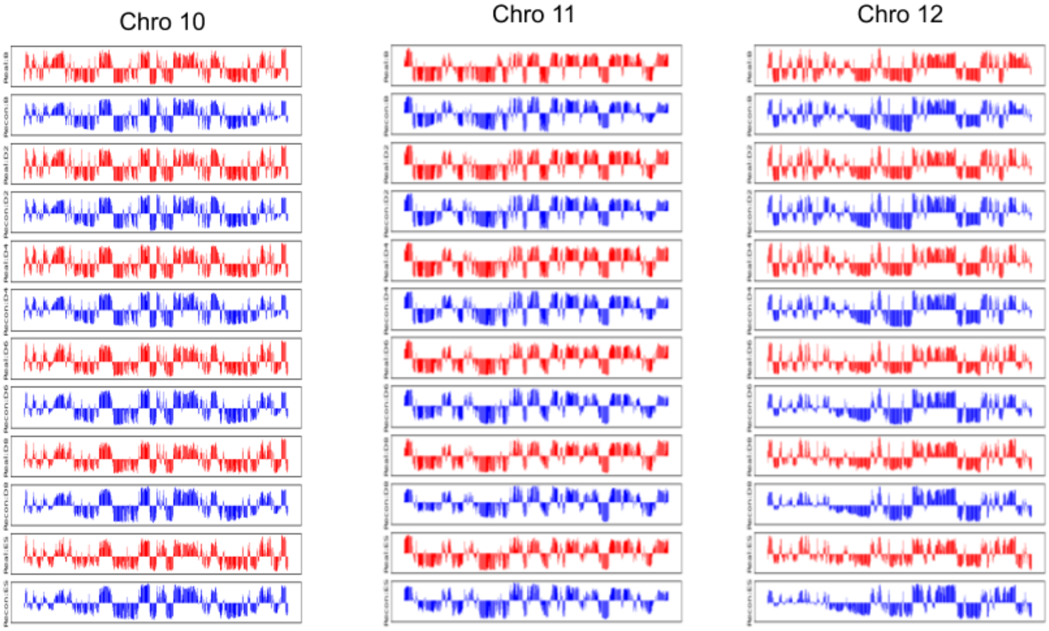
Chro 10
Chro 11
Chro 12

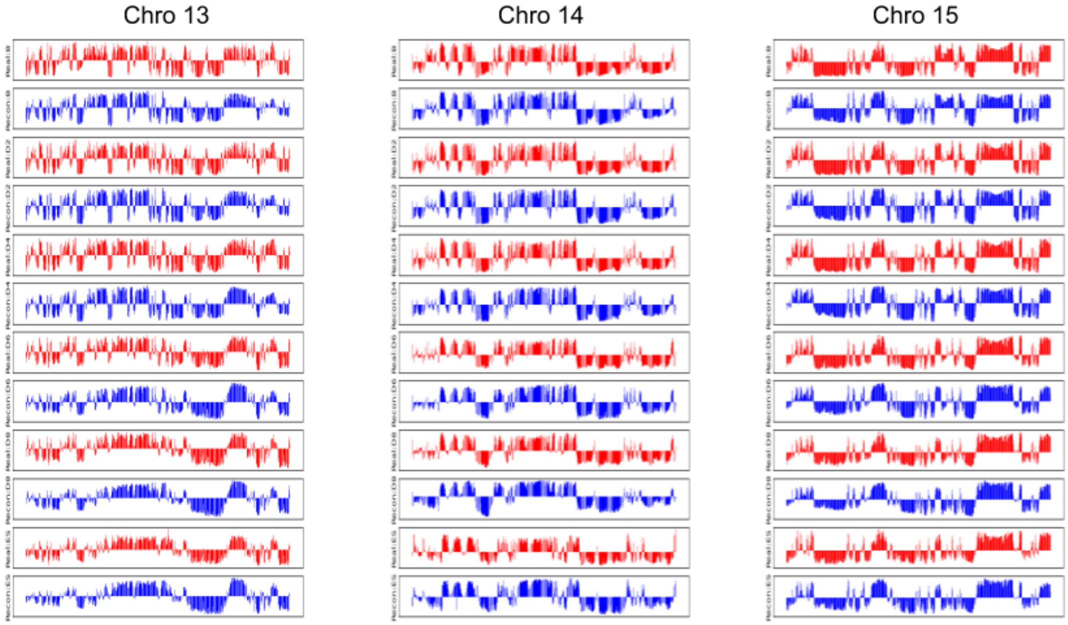

Chro 13
Chro 14
Chro 15
Real:B
Recon:B
Real:D2
Recon:D2
Real:D4
Recon:D4
Real:D6
Recon:D6
Real:D8
Recon:D8
Real:ES
Recon:ES

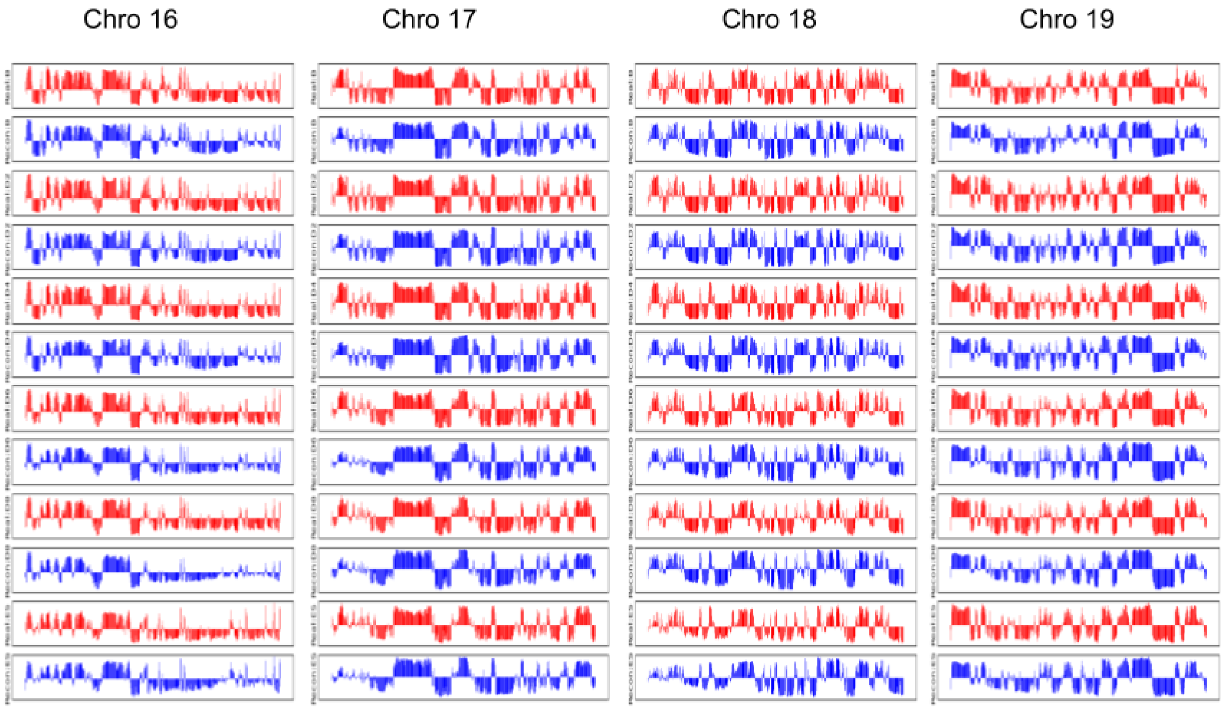

Figure S13 Pearson Mats

Plots of AB compartment assignments (red) biological Hi-C (blue) reconstructed Hi-C contacts.

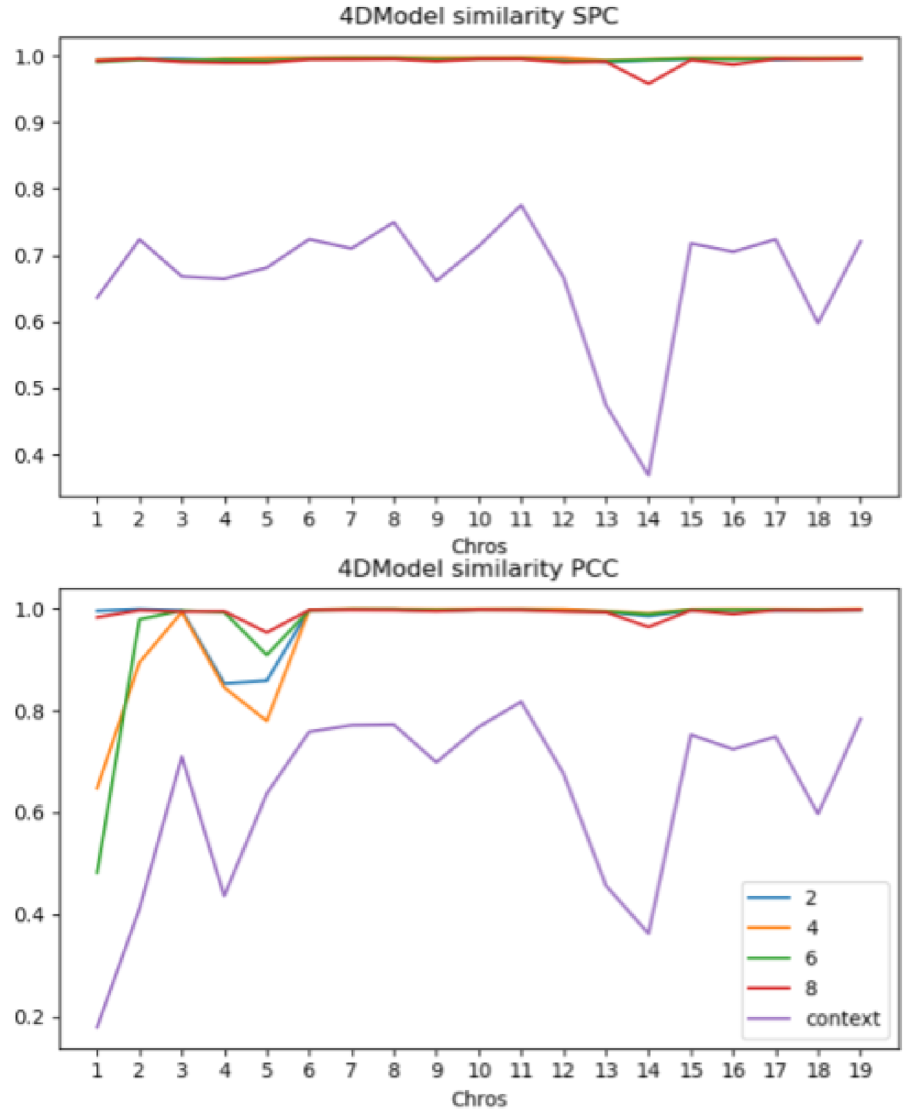

Figure S14 4D Model Similarity of Interpolated iPSC and Full iPSC Models

Avg spearman and pearson correlation between distance vectors of structural conformation at all time points of full cardiomyocyte structures and interpolation structures. The context metrics show the correlation between distance vectors for the structural conformation at the beginning and end of an interpolation structure. This indicated consistently higher similarity between two 4D structures at each time than between a 4D structures starting and ending conformation.

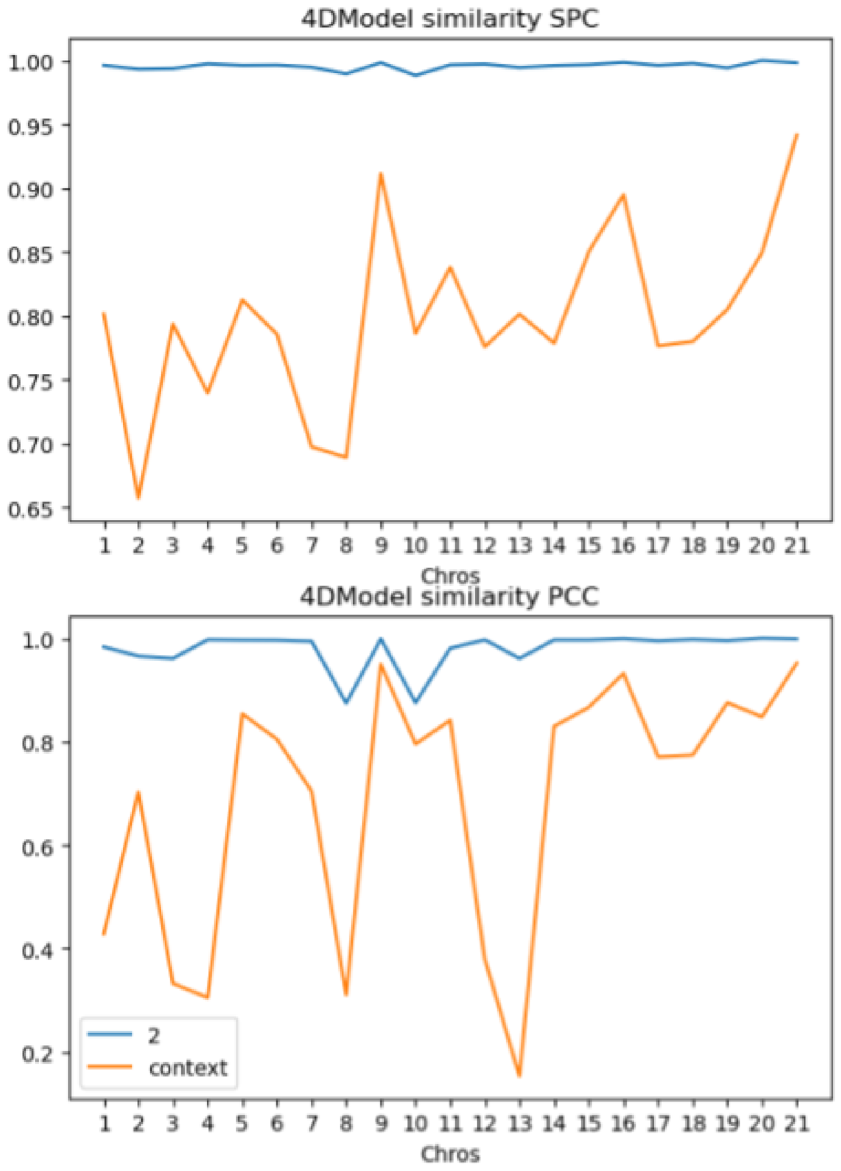

Figure S15 4D Model Similarity of Interpolated Cardio and Cull Cardio

Avg spearman and pearson correlation between distance vectors of structural conformation at all time points of full cardiomyocyte structures and interpolation structures. The context metrics show the correlation between distance vectors for the structural conformation at the beginning and end of an interpolation structure. This indicated consistently higher similarity between two 4D structures at each time than between a 4D structures starting and ending conformation.

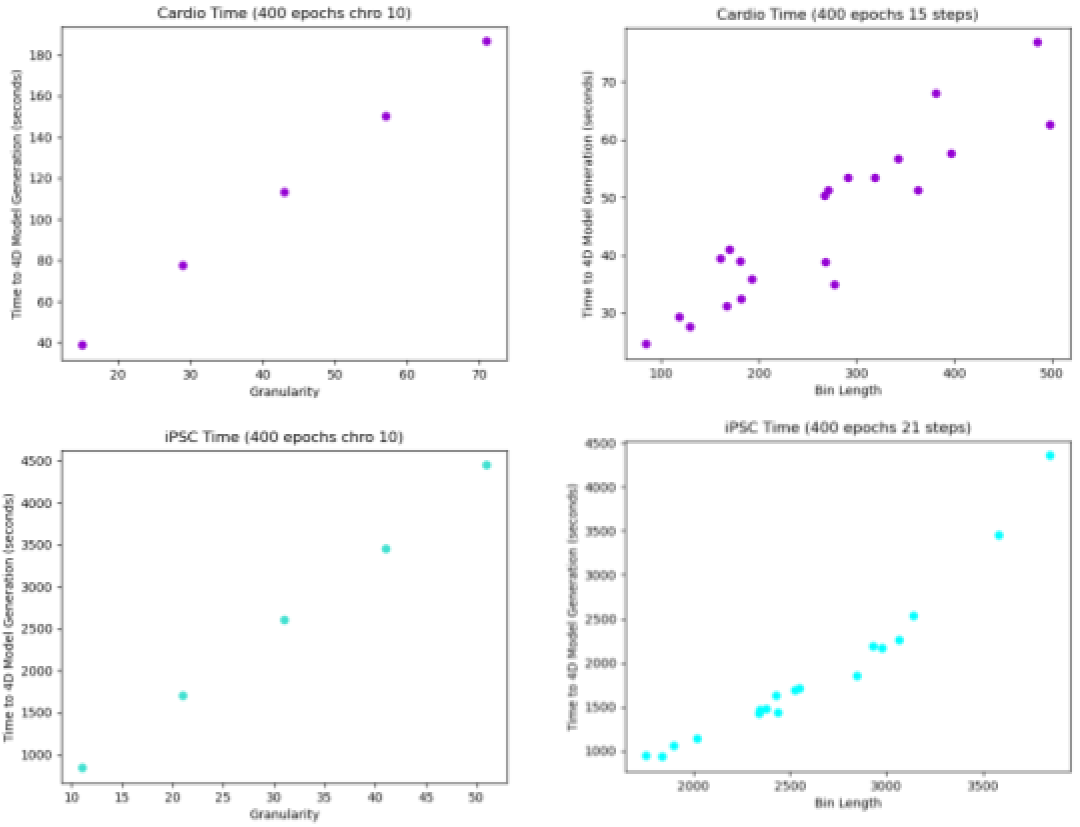
Cardio Time (400 epochs chro 10)
Time to 4D Model Generation (seconds)
Granularity
Cardio Time (400 epochs 15 steps)
Time to 4D Model Generation (seconds)
Bin Length
iPSC Time (400 epochs chro 10)
Time to 4D Model Generation (seconds)
Granularity
iPSC Time (400 epochs 21 steps)
Time to 4D Model Generation (seconds)
Bin Length

Figure S16 Run Time

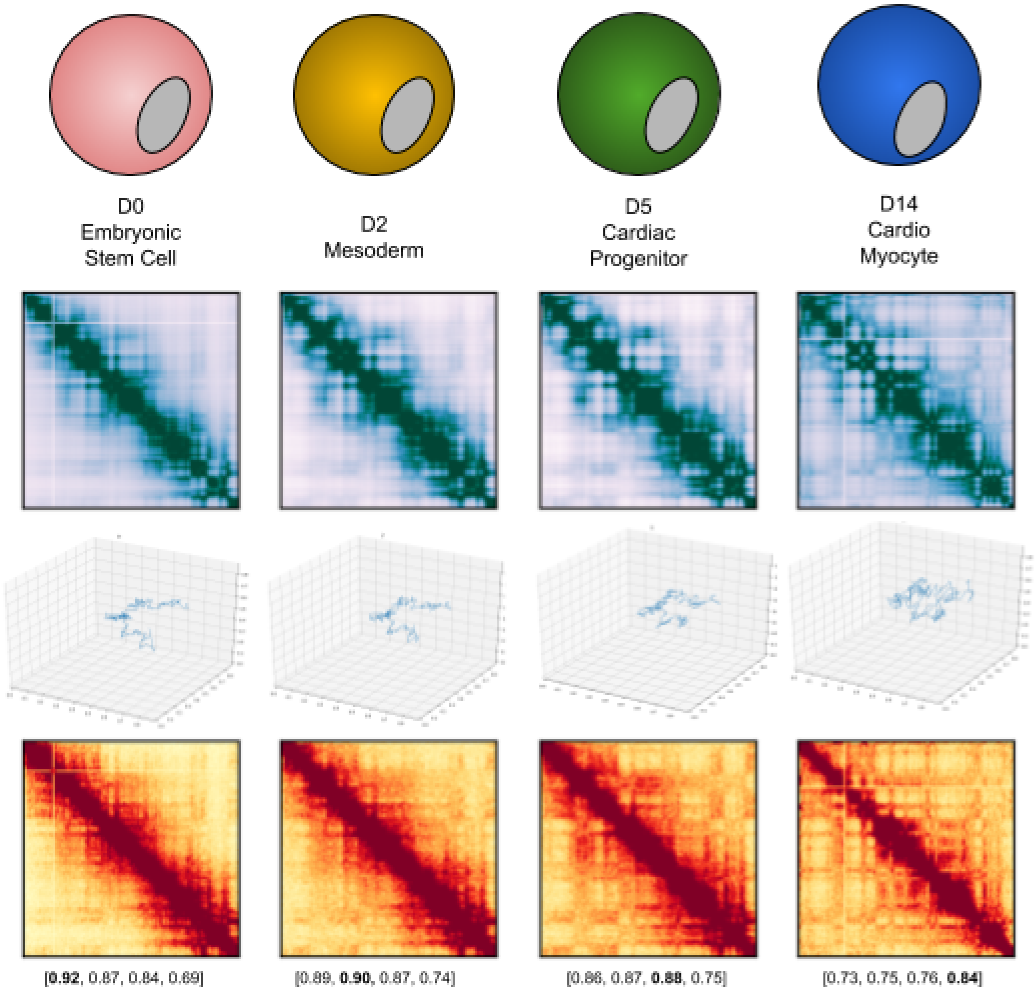

Figure S17 Cardio Schematic

Chromosome 13. (a) Days Hi-C experiments are taken. (b) Hi-C contact matrices © reconstructed structure (4) Reconstructed Contact maps

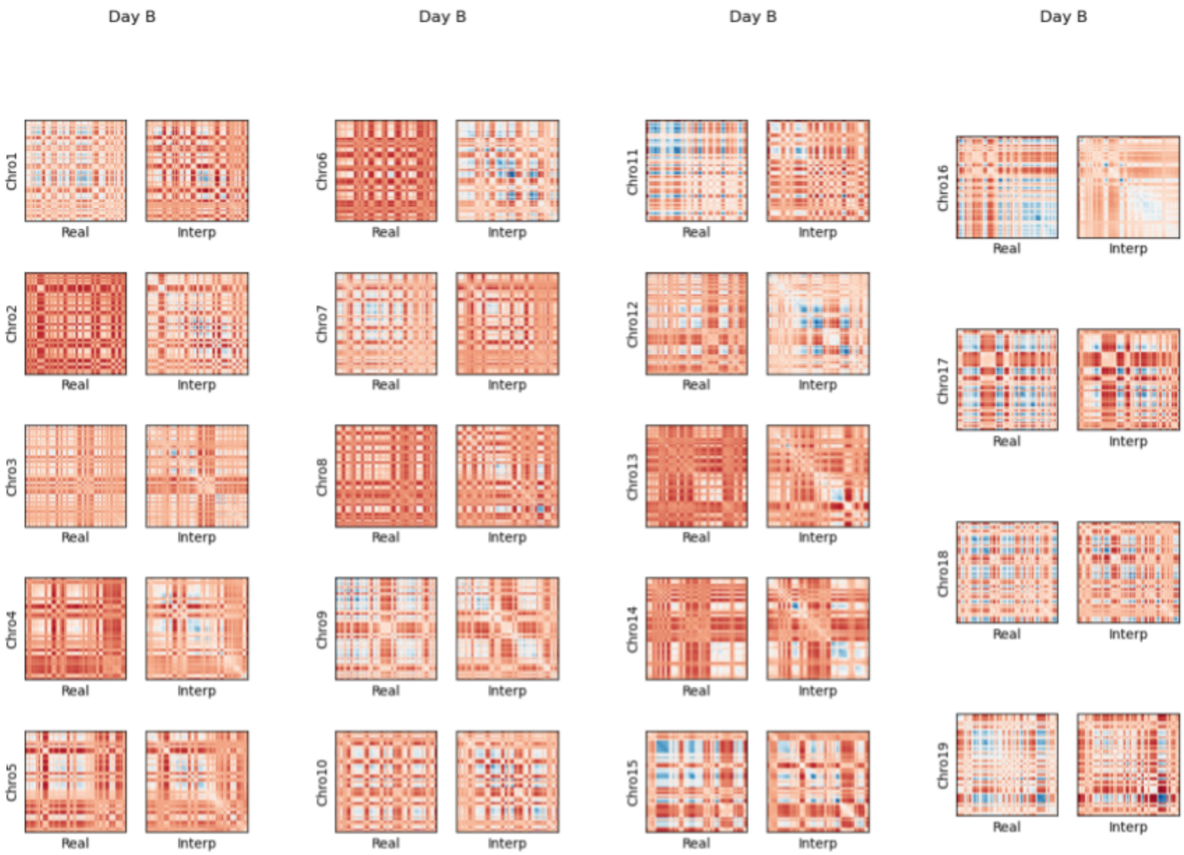

Day B
Day B
Day B
Day B
Chro1
Chro2
Chro3
Chro4
Chro5
Chro6
Chro7
Chro8
Chro9
Chro10
Chro11
Chro12
Chro13
Chro14
Chro15
Chro16
Chro17
Chro18
Chro19
Real
Interp

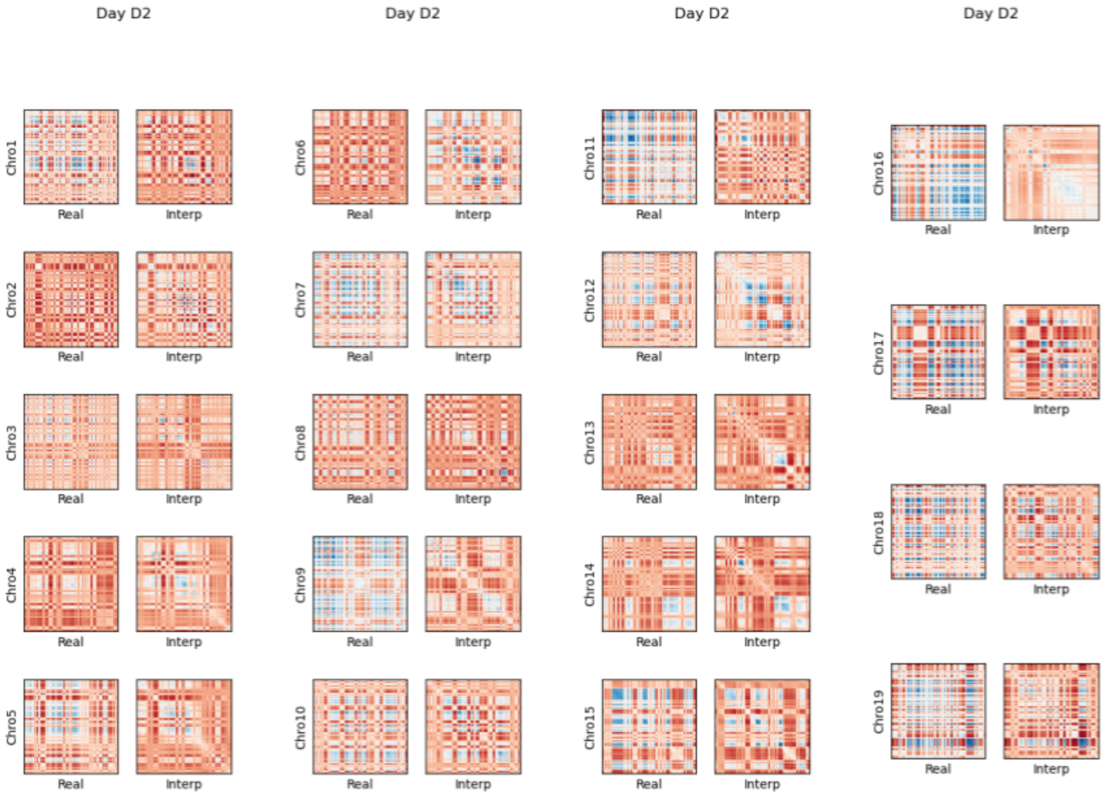

Day D2
Day D2
Day D2
Day D2
Chro1
Chro2
Chro3
Chro4
Chro5
Chro6
Chro7
Chro8
Chro9
Chro10
Chro11
Chro12
Chro13
Chro14
Chro15
Chro16
Chro17
Chro18
Chro19
Real
Interp

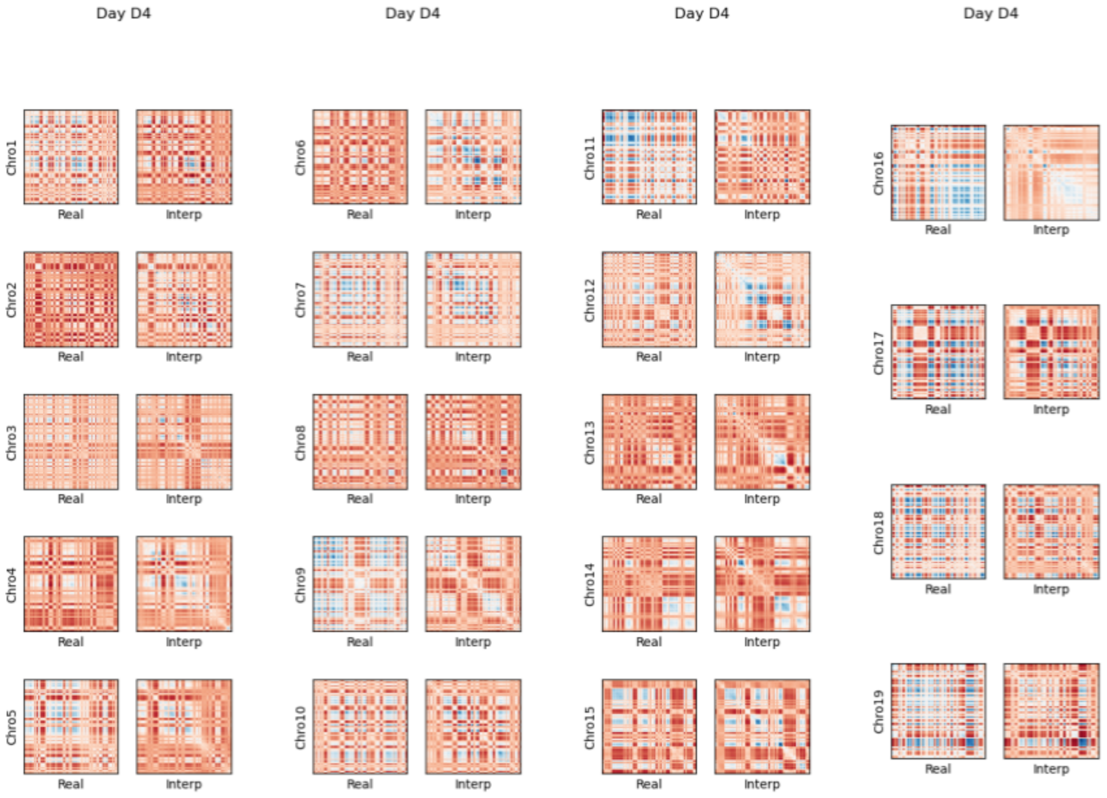

Day D4
Day D4
Day D4
Day D4
Chro1
Chro2
Chro3
Chro4
Chro5
Chro6
Chro7
Chro8
Chro9
Chro10
Chro11
Chro12
Chro13
Chro14
Chro15
Chro16
Chro17
Chro18
Chro19
Real
Interp

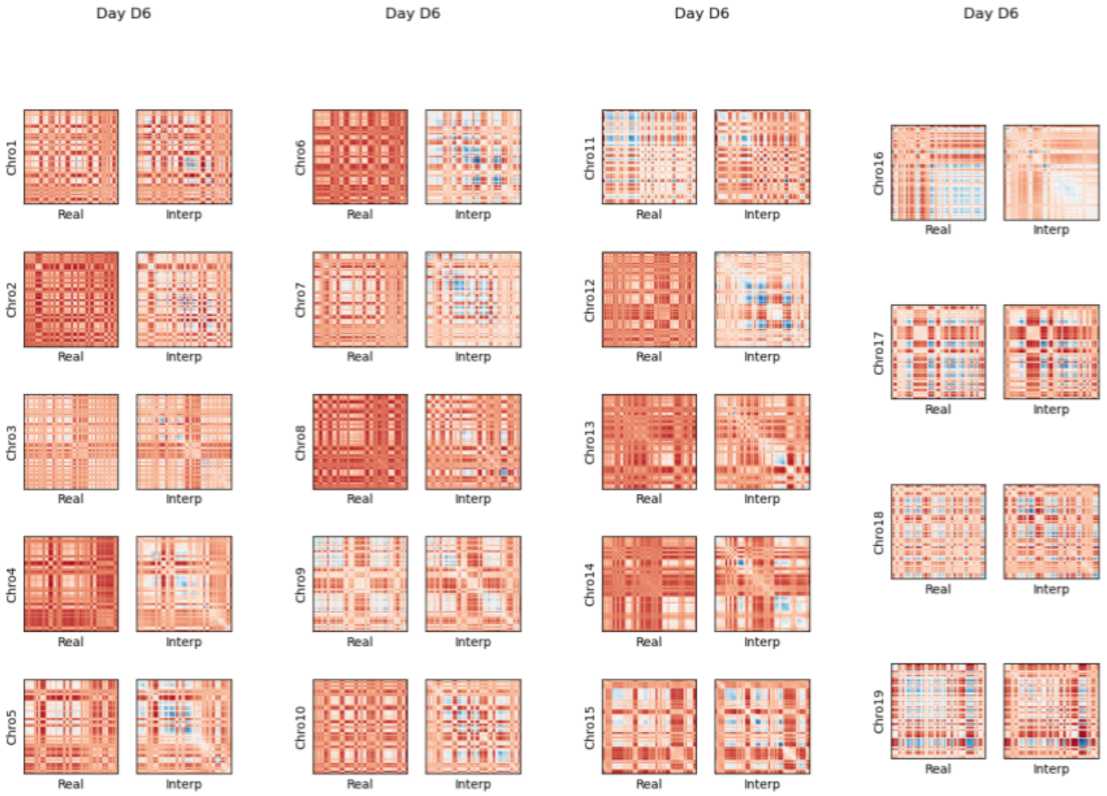
Day D6
Day D6
Day D6
Day D6
Chro1
Chro2
Chro3
Chro4
Chro5
Chro6
Chro7
Chro8
Chro9
Chro10
Chro11
Chro12
Chro13
Chro14
Chro15
Chro16
Chro17
Chro18
Chro19
Real
Interp

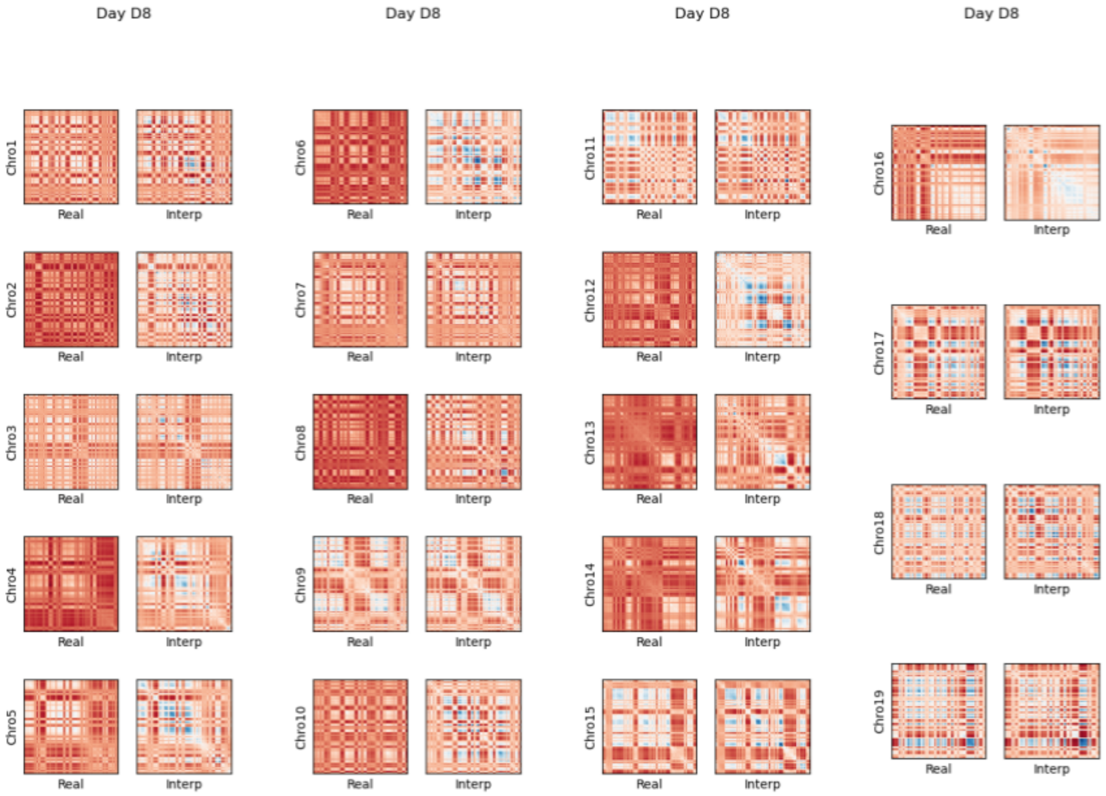

Day D8
Day D8
Day D8
Day D8
Chro1
Chro2
Chro3
Chro4
Chro5
Chro6
Chro7
Chro8
Chro9
Chro10
Chro11
Chro12
Chro13
Chro14
Chro15
Chro16
Chro17
Chro18
Chro19
Real
Interp

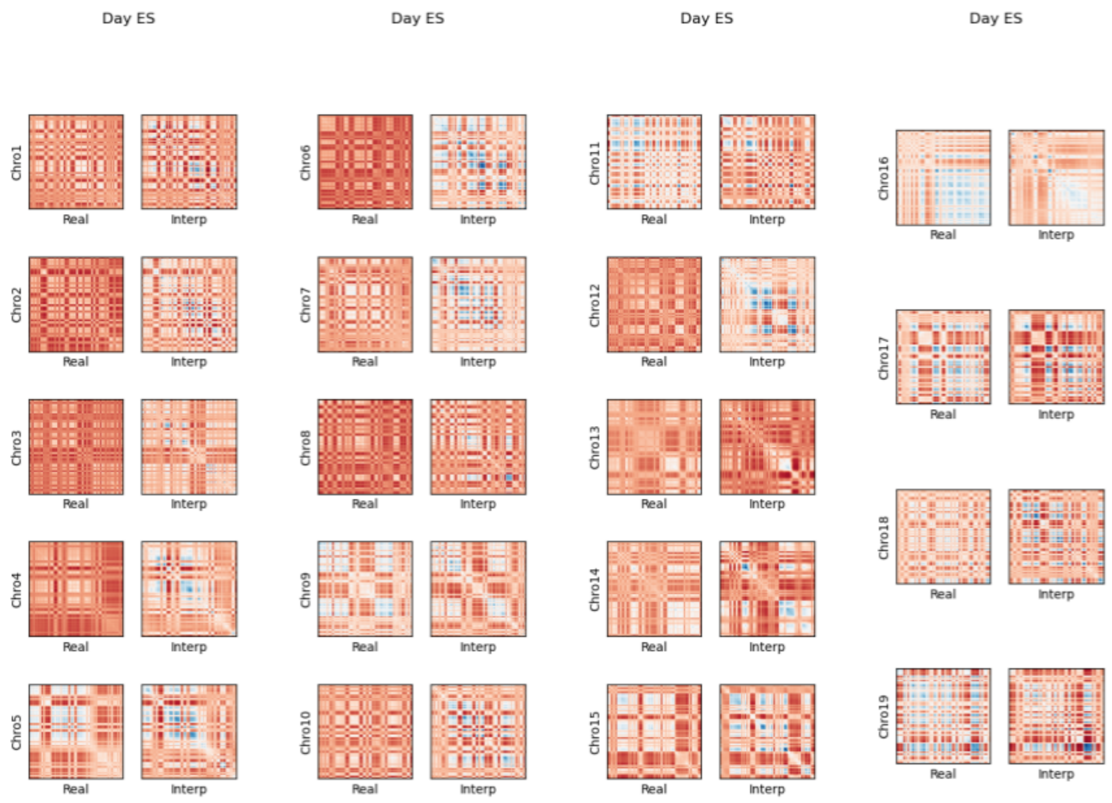

Figure S18 AB Vec Pearson Matrices

AB compartments Pearson correlation matrices for (left) real Hi-C and (right) reconstructed Hi-C data.

# eta	lr	alpha	avg_ pcc
1	1.00E-01	0.6	-0.0317
1	1.00E-01	0.8	-0.0358
1	1.00E-01	1	-0.038
1	1.00E-03	0.6	-0.0017
1	1.00E-03	0.8	-0.0035
1	1.00E-03	1	-0.0071
1	1.00E-04	0.6	0.7492
1	1.00E-04	0.8	0.7162
1	1.00E-04	1	0.6774
1	1.00E-05	0.6	0.354
1	1.00E-05	0.8	0.4005
1	1.00E-05	1	0.4323
1000	1.00E-01	0.6	nan
1000	1.00E-01	0.8	nan
1000	1.00E-01	1	nan
1000	1.00E-03	0.6	-0.2323
1000	1.00E-03	0.8	-0.1823
1000	1.00E-03	1	-0.1452
1000	1.00E-04	0.6	0.7493
1000	1.00E-04	0.8	0.7166
1000	1.00E-04	1	0.6769
1000	1.00E-05	0.6	0.3546
1000	1.00E-05	0.8	0.4209
1000	1.00E-05	1	0.4741
1000000	1.00E-01	0.6	nan
1000000	1.00E-01	0.8	nan
1000000	1.00E-01	1	nan
1000000	1.00E-03	0.6	nan
1000000	1.00E-03	0.8	nan
1000000	1.00E-03	1	nan
1000000	1.00E-04	0.6	nan
1000000	1.00E-04	0.8	nan

1000000	1.00E-04	1	nan
1000000	1.00E-05	0.6	0.252
1000000	1.00E-05	0.8	0.2787
1000000	1.00E-05	1	0.2924

Figure S19 Hyper Parameter Tuning

# Day	Chro	Numerical Interp	4DMax recon	4DMax interp
D2	1	0.734	0.798	0.777
D4	1	0.712	0.733	0.732
D6	1	0.663	0.692	0.693
D2	2	0.751	0.813	0.785
D4	2	0.727	0.762	0.758
D6	2	0.685	0.733	0.727
D2	3	0.756	0.816	0.798
D4	3	0.733	0.771	0.76
D6	3	0.685	0.733	0.726
D2	4	0.764	0.825	0.794
D4	4	0.742	0.773	0.769
D6	4	0.693	0.74	0.721
D2	5	0.772	0.832	0.804
D4	5	0.747	0.781	0.773
D6	5	0.693	0.738	0.712
D2	6	0.748	0.814	0.785
D4	6	0.729	0.766	0.761
D6	6	0.677	0.726	0.699
D2	7	0.775	0.83	0.8
D4	7	0.748	0.78	0.769
D6	7	0.698	0.743	0.703
D2	8	0.764	0.827	0.801
D4	8	0.741	0.78	0.772
D6	8	0.682	0.738	0.697
D2	9	0.763	0.823	0.797
D4	9	0.732	0.77	0.763
D6	9	0.667	0.731	0.692
D2	10	0.764	0.828	0.802
D4	10	0.739	0.777	0.77
D6	10	0.688	0.748	0.72
D2	11	0.791	0.844	0.813
D4	11	0.764	0.797	0.785

D6	11	0.716	0.767	0.741
D2	12	0.746	0.815	0.776
D4	12	0.715	0.756	0.743
D6	12	0.661	0.721	0.681
D2	13	0.716	0.779	0.742
D4	13	0.689	0.72	0.712
D6	13	0.639	0.7	0.674
D2	14	0.732	0.808	0.774
D4	14	0.707	0.752	0.744
D6	14	0.645	0.712	0.677
D2	15	0.781	0.834	0.802
D4	15	0.76	0.792	0.78
D6	15	0.709	0.762	0.704
D2	16	0.789	0.842	0.807
D4	16	0.773	0.8	0.786
D6	16	0.721	0.769	0.718
D2	17	0.79	0.837	0.803
D4	17	0.771	0.796	0.784
D6	17	0.712	0.752	0.702
D2	18	0.766	0.816	0.782
D4	18	0.737	0.772	0.751
D6	18	0.673	0.73	0.658
D2	19	0.802	0.835	0.794
D4	19	0.791	0.808	0.781
D6	19	0.746	0.781	0.702

Figure S20 Performance Comparison to Numerical Interpolation

Videos

Videos are stored as gif files and are available:
http://sysbio.rnet.missouri.edu/3dgenome/4DMax/

S1 Video Collection 1 Synthetic Videos

http://sysbio.rnet.missouri.edu/3dgenome/4DMax/Movies%201%20Synthetic.zip

S2 Video Collection 2 iPSC Videos

http://sysbio.rnet.missouri.edu/3dgenome/4DMax/Movies%202%20iPSC%20Full.zip

S3 Video Collection 3 Cardio Videos

http://sysbio.rnet.missouri.edu/3dgenome/4DMax/Movies%203%20Cardio%20Videos.zip

S4 Video Collection 4 iPSC Missing Chros

http://sysbio.rnet.missouri.edu/3dgenome/4DMax/Movies%204%20iPSC%20Missing.zip

S5 Video Collection 5 Cardio Missing Chros

http://sysbio.rnet.missouri.edu/3dgenome/4DMax/Movies%205%20Cardio%20Missing%20Chros.zip

S6 Video Collection 6 Changing Resolution

http://sysbio.rnet.missouri.edu/3dgenome/4DMax/Movies%206%20Changing%20Resolution.zip

S7 Video Collection 7 Changing Granularity

http://sysbio.rnet.missouri.edu/3dgenome/4DMax/Movies%207%20Changin%20Granularity.zip

S8 Video Collection 8 Changing Granularity

http://sysbio.rnet.missouri.edu/3dgenome/4DMax/Movies%208%20Hyper%20Param.zip
